# Integration of Signals along Orthogonal Axes of the Vertebrate Neural Tube Controls Progenitor Competence and Increases Cell Diversity

**DOI:** 10.1371/journal.pbio.1001907

**Published:** 2014-07-15

**Authors:** Noriaki Sasai, Eva Kutejova, James Briscoe

**Affiliations:** Division of Developmental Biology, Medical Research Council, National Institute for Medical Research, London, United Kingdom; University of Dundee, United Kingdom

## Abstract

A relatively small number of signals are responsible for the variety and pattern of cell types generated in developing embryos. In part this is achieved by exploiting differences in the concentration or duration of signaling to increase cellular diversity. In addition, however, changes in cellular competence—temporal shifts in the response of cells to a signal—contribute to the array of cell types generated. Here we investigate how these two mechanisms are combined in the vertebrate neural tube to increase the range of cell types and deliver spatial control over their location. We provide evidence that FGF signaling emanating from the posterior of the embryo controls a change in competence of neural progenitors to Shh and BMP, the two morphogens that are responsible for patterning the ventral and dorsal regions of the neural tube, respectively. Newly generated neural progenitors are exposed to FGF signaling, and this maintains the expression of the Nk1-class transcription factor Nkx1.2. Ventrally, this acts in combination with the Shh-induced transcription factor FoxA2 to specify floor plate cells and dorsally in combination with BMP signaling to induce neural crest cells. As development progresses, the intersection of FGF with BMP and Shh signals is interrupted by axis elongation, resulting in the loss of Nkx1.2 expression and allowing the induction of ventral and dorsal interneuron progenitors by Shh and BMP signaling to supervene. Hence a similar mechanism increases cell type diversity at both dorsal and ventral poles of the neural tube. Together these data reveal that tissue morphogenesis produces changes in the coincidence of signals acting along orthogonal axes of the neural tube and this is used to define spatial and temporal transitions in the competence of cells to interpret morphogen signaling.

## Abstract 2

FGF gates competence to generate Floor Plate and Neural Crest in response to Shh and BMP signals by controlling expression of the transcription factor Nkx1.2.

## Author Summary

During embryonic development different cell types arise at different times and places. This diversity is produced by a relatively small number of signals and depends, at least in part, on changes in the way cells respond to each signal. One example of this so-called change in “competence” is found in the vertebrate spinal cord where a signal, Sonic Hedgehog (Shh), induces a glial cell type known as floor plate (FP) at early developmental times, while the same signal later induces specific types of neurons. Here, we dissected the molecular mechanism underlying the change in competence, and found that another signal, FGF, is involved through its control of the transcription factor Nkx1.2. In embryos, Shh and FGF are produced perpendicular to one another and FP is induced where the two signals intersect. The position of this intersection changes as the embryo elongates and this determines the place and time FP is produced. A similar strategy also appears to apply to another cell type, neural crest. In this case, the intersection of FGF with BMP signal is crucial. Together the data provide new insight into the spatiotemporal control of cell type specification during development of the vertebrate spinal cord.

## Introduction

A large array of distinct cell types is generated during embryonic development in response to a relatively small number of inductive signals. A mechanism to explain this was described by C.H. Waddington in his influential book “Organizers and Genes” [1]. In this work he proposed that the specification of cell identity resulted from an interplay between “evocators,” extrinsic inductive signals, and the specific intrinsic response of the tissue to the inductive signal, which he termed “competence.” In this view inductive signals initiate cellular differentiation but the fate induced by the signal is intrinsic to the responding cell. Thus temporal shifts in a cell's competence provide a means to increase the diversity of cell types induced while maintaining control over the pattern in which they are generated.

One example where this is relevant is the development of the vertebrate nervous system. In the spinal cord, this involves the well-ordered generation of a large variety of molecularly distinct cell types including the neurons that process sensory information and control motor movement and the migratory neural crest cells (NCCs) that form the peripheral nervous system [2]–[6]. The ventral part of the spinal cord contains motor neurons (MNs) and interneurons (V0–V3) as well as the morphologically distinct nonneuronal cells of the floor plate (FP) [6]. These cell types are produced from domains of progenitors arrayed along the dorsal ventral axis, each of which is defined by the expression of transcription factors including Olig2 (pMN), Nkx2.2 (p3), and Arx (FP) [7]–[10]. By contrast, NCCs and dI1–dI3 interneurons [3] are produced in the dorsal neural tube. Similar to the ventral neural tube, the progenitors of these cell types can be distinguished by their distinct gene expression programmes—Snail2 and Sox10 in NCCs and Olig3 in dI1–3 progenitors [11]–[13].

The stereotypic organization of neural tube cell types depends on secreted factors. Sonic Hedgehog (Shh), emanating from the FP and the underlying notochord, is involved in patterning the ventral neural tube [14]. The dorsal neural tube is patterned by a distinct set of signals, prominent amongst these are members of the TGFβ family [15]. Several studies indicate that both dorsal and ventral signals function as morphogens to regulate differential gene expression in a graded manner [16]–[18]. Nevertheless a simple morphogen mechanism does not appear sufficient to explain the entirety of cell diversity produced by these factors. Importantly, the time at which cells are exposed to Shh or BMP has a significant influence over the cell types generated. For example, the induction of FP cells, which are situated in the most ventral part of the neural tube, require exposure to Shh at an early developmental time point [10],[19]. Accordingly, progenitors exposed to similar amounts of Shh but at later developmental times differentiate into p3 progenitors of V3 neurons instead of FP cells [10],[19]. Likewise, the differentiation of NCCs depends on the time-specific exposure to dorsal signals [20]. Neural cells exposed to BMP4/7 at early time points differentiate into the NCCs, whereas neural cells exposed to the same signals at later time points differentiate into the dorsal interneurons [20].

How neural cells change their competence to inductive signals over developmental time is unclear. It is notable, however, that when first generated in the posterior neural plate, neural progenitors are exposed to FGF signaling, but as development proceeds axis elongation interrupts FGF signaling and progenitors are exposed to retinoic acid (RA) secreted from the adjacent somites [21]. The switch from FGF to RA signaling has been suggested to control the timing of neuronal differentiation in the spinal cord [22],[23]. Moreover, the repression by FGF signaling of Pax6, Irx3, and other transcription factors expressed in neural progenitors has been suggested to contribute to the maintenance of the undifferentiated state [21]. Whether this state provides cells with the competence to generate FP and NCC in response to appropriate inductive signals is unclear.

Here we investigate the shift in generation from FP to p3 and from NCCs to dorsal interneurons to identify the mechanisms responsible for the change in competence. We provide evidence that FGF signaling, in early neural progenitors, provides cells with the competence to differentiate into FP and NCCs in response to Shh and BMP, respectively. Furthermore, we find that Nkx1.2, a NK-1 transcription factor, which is regulated by FGF signaling [24], mediates this competence and represses the expression of Pax6 and Irx3 [8],[21],[25]. In the case of FP, the coincidence of Nkx1.2 with Shh-induced FoxA2 expression defines the domain in which the FP will differentiate. Subsequently, axis elongation and the ensuing decline in FGF signaling result in the down-regulation of Nkx1.2 expression. This then allows cells to generate ventral and dorsal interneuron progenitors in response to Shh and BMP signaling. Hence the dynamics of cell movement drive temporal changes in signaling and gene expression in neural progenitors and these in turn control the transcription network that determines the intrinsic competence of cells to respond to morphogens acting along the orthogonal axis. Together the data reveal a molecular mechanism in which the interplay between cell competence and inductive signals increases the diversity cell types in the neural tube and determines their pattern of generation.

## Results

### FGF Provides Competence for FP Induction

**Figure 1:**
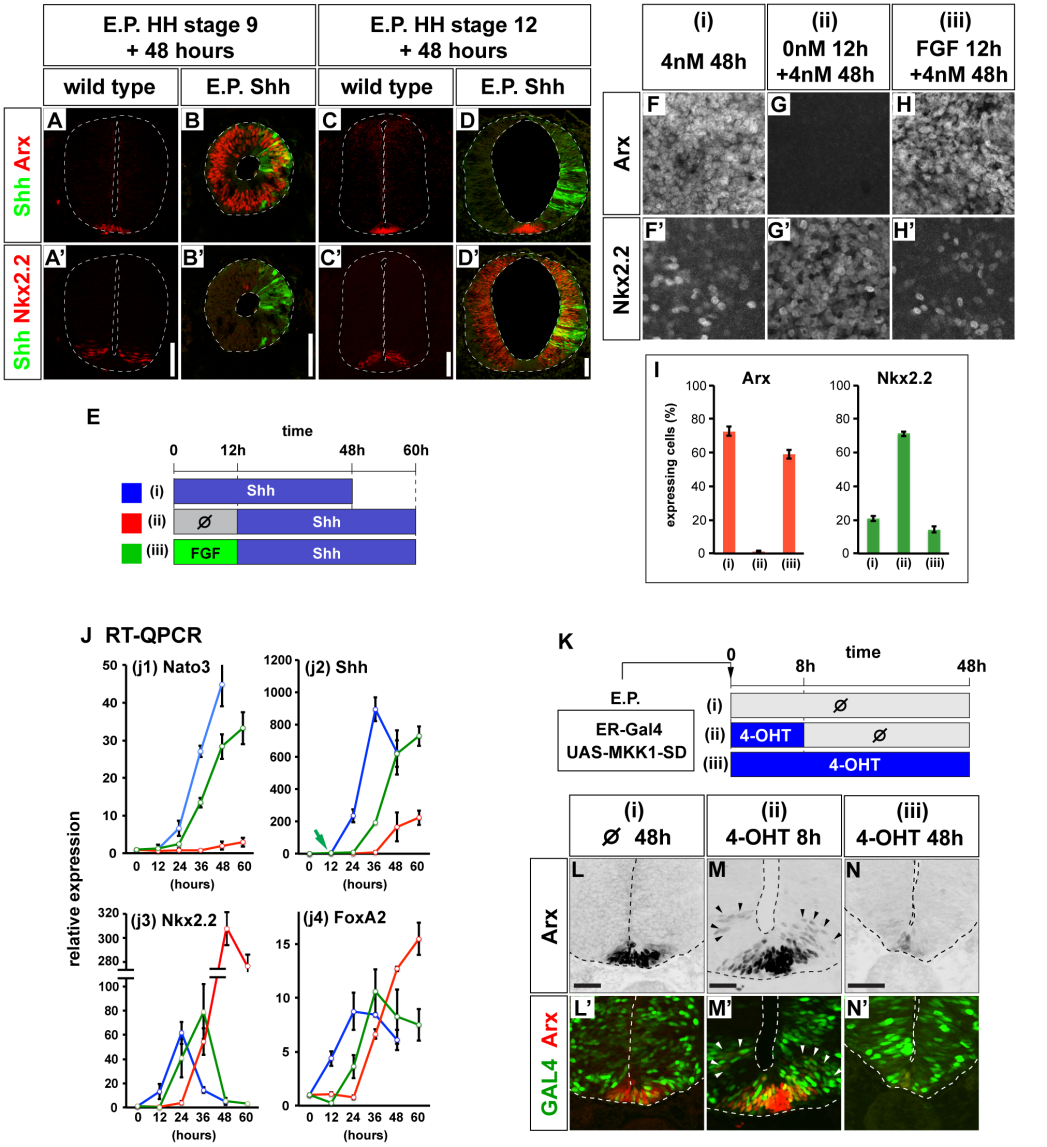
FGF regulates the competence for FP induction by Shh. (A–D′) Early exposure of neural progenitors to Shh is necessary for FP induction in vivo. HH stage 9 (B, B′) or HH stage 12 (D, D′) embryos were electroporated with an expression plasmid encoding the amino-terminal signaling peptide of Shh (Shh-N). Electroporated (B, B′, D, D′) and control embryos (A, A′, C, C′) were harvested at 48 hpt. FP and p3 induction was assayed with immunohistochemisty on sections of neural tube for Arx (A, B, C, D), Nkx2.2 (A′, B′, C′, D′), and GFP (green cells in panels in A–D′). Scale bar (A′, B′, C′, D′) = 50 µm. (E–I) FGF prolongs the competence period for FP induction. (E) Schematic representation of the explant experiment in (F–J). (F–I) Representative images (F–H′) and quantitation (I) of explants treated with the indicated conditions assayed for Nkx2.2 and Arx expression. (J) Expression levels of the indicated genes assayed by qRT-PCR. Color code corresponds to the experimental conditions shown in (E). (K–N′) Transient activation of FGF signaling promotes FP differentiation. The expression vector pCIG-ER-Gal4-VP16 (containing a GFP gene) together with a plasmid containing HA-MKK-SD driven by 14×UAS were transfected into the HH stage 8 embryos. Embryos were exposed to 4-hydroxytamoxifen (4-OHT) for 8 h (ii) or for 48 h (iii) and assayed at 48 h. (L–N′) Ectopic Arx expression was present only after transient induction of HA-MKK-SD (ii; M, M′; arrowheads); prolonged activation of HA-MKK-SD inhibited Arx expression (iii; N, N′). GFP expression marks transfected cells (L′, M′, N′). Scale bar (L, M, N) = 50 µm.

We previously showed that early exposure to Shh is required for neural progenitors to induce FP, characterized by Arx expression (Figure 1A) [10]. Forced expression of Shh, by in ovo electroporation, in the early [Hamburger Hamilton (HH) stage 9] [26] neural tube resulted in the broad ectopic induction of FP 48 h posttransfection (hpt) both in vivo (12/15; Figure 1A,B) and in vitro (Figure S1). By contrast, forced expression of Shh later (HH stage 12) did not induce ectopic FP (0/10; Figure 1C,D). Instead, progenitors expressed Nkx2.2, characteristic of p3 neural progenitors that are normally situated in a progenitor domain dorsal and adjacent to the FP (Figures 1A′,B′,C′,D′ and S2B,C) were induced. Moreover, longer incubation (72 h) of embryos transfected at HH stage 12 with Shh did not lead to induction of Arx (0/8; Figure S2G,H′). Similar results were obtained assaying Nato3 [27] and Nkx6.1 [28], which are expressed in FP and p2–p3 domains, respectively (unpublished data). Thus, neural progenitors lose their competence to generate FP in response to Shh between HH stage 9 and HH stage 12. A similar change in competence was observed in ex vivo experiments. Intermediate [i] neural plate explants from HH stage 10 embryos treated with 4 nM Shh for 48 h expressed Arx in most of the cells (Figure 1E,F,F′,I) [10]. By contrast, explants that were incubated in the absence of Shh for 12 h before the addition of 4 nM Shh induced little if any Arx expression (Figure 1E,G,I). Instead Nkx2.2 expression was maintained in these explants (Figure 1G′,I). A longer culture, up to 72 h, did not change the expression profile (unpublished data).

The timing of the change in FP competence led us to focus on signals present in the neural plate of HH stage 10 embryos. To this end we tested the function of FGF [21], Wnt, and RA [29] signaling in HH stage 10 [i] explants. Culturing [i] explants in the presence of Wnt or the RA inhibitor BMS493 for 12 h before replacing with media containing 4 nM Shh did not result in the induction of FP (see Materials and Methods; unpublished data). By contrast, FP gene induction was observed if [i] explants were transiently exposed to 5 nM bFGF for 12 h in the absence of Shh and then transferred to 4 nM Shh for 48 h (Figure 1E,H,I). Moreover, additional markers of the FP identity including *Nato3* [Figure 1J(j1)] [27], *Shh* [Figure 1J(j2)] [10], *HES1* [30], and FoxP2 [31] were restored by this treatment (Figure 1J; unpublished data). In contrast, expression of the p3 marker *Nkx2.2* decreased [Figure 1H′,I,J(j3)]. The expression of *FoxA2* was similar in early and FGF-treated conditions (Figures 1J(j4) and S2D–F). FGF on its own did not induce Arx expression (unpublished data) at 48 h, nor did FGF induce *Shh* gene expression at 12 h [green arrow in Figure 1J(j2)] or Shh signaling (green arrow in Figure S2I), indicating that FGF on its own is not sufficient to induce FP identity.

We next asked whether FGF promotes FP differentiation in the presence of Shh in vivo. Surprisingly, the sustained expression of FGF8b in vivo, using in ovo electroporation, eliminated Arx expression and resulted in the ventral expansion of Nkx2.2 expression (5/5; Figure S2J–K′). In light of the posteriorly restricted expression of FGF8 in vivo [21] we speculated that transient FGF signaling might be necessary for FP differentiation. To test this hypothesis we took advantage of a regulatable expression system to activate the FGF pathway in vivo for a limited time period [32]. We transfected a constitutively active version of the FGF-activated MAP kinase, MKK1 (HA-MKK1-SD) [33], under the control of a Tamoxifen-regulated Gal4 transactivator (ER-Gal4-VP16). Following electroporation at HH stage 8+, we stimulated HA-MKK1-SD using Tamoxifen for 8 h at HH stage 10. The drug was then thoroughly washed out, and the embryos were cultured for an additional 40 h (Figure 1K,M,M′). This resulted in a transient up-regulation of luciferase activity (Figure S2L) and HA-MKK1-SD expression (Figure S2M–Q′). Moreover transient activation of MKK1 resulted in an expansion of Arx expression [6/8; Figure 1K(ii),M,M′]. This suggested that transient, but not sustained, FGF signaling prolonged the competence period for FP induction and this facilitated the expanded FP induction in response to the increasing amplitude of the Shh gradient. Embryos without Tamoxifen did not show an expansion of Arx [0/6; Figure 1K(i),L,L′], and sustained treatment with Tamoxifen abolished FP induction, consistent with the unregulated activation of FGF [6/8; Figures 1K(iii),N,N′ and S2J–K′,W,W′,Z,Z′). Together these data indicate that transient exposure of neural progenitors to FGF provides competence for neural progenitors to differentiate into the FP in response to Shh.

### FGF Signalling Is Necessary for FP Induction

**Figure 2:**
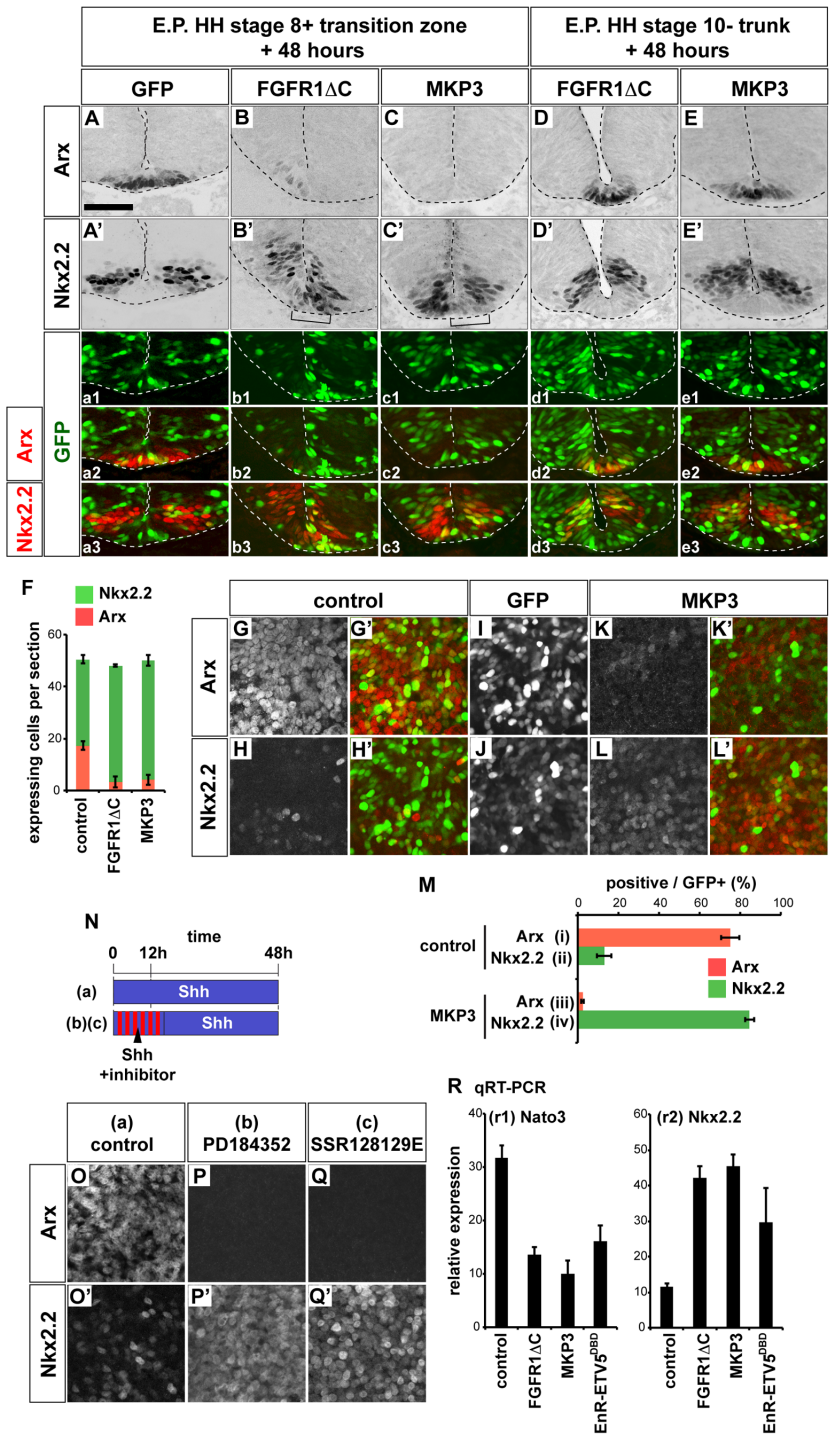
FGF is necessary for FP induction. (A–E′, a1–e3) Blocking FGF signaling inhibits FP differentiation in vivo in a stage-dependent manner. Constructs expressing control GFP (A, A′, a1–a3), FGFR1ΔC (B, B′, b1–b3, D, D′, d1–d3), or MKP3 (C, C′, c1–c3, E, E′, e1–e3) were electroporated at HH stage 8+ (A-c3) or at HH stage 10- (D-e3) and embryos were incubated for 48 h. The expression of GFP (green in a1–e3), Arx (A, B, C, D, E, red in a2, b2, c2, d2, e2), and Nkx2.2 (A′, B′, C′, D′, E′, red in a3, b3, c3, d3, e3) was analyzed by immunohistochemistry. Affected cells are indicated with brackets (B′, C′). Scale bar (A) for (A–E′, a1–e3) = 50 µm. (F) Quantification in affected embryos of cells expressing Arx or Nkx2.2 in (A–C). (G–R) FGF signaling is necessary for FP differentiation in vitro. (G–M) [i] explants that had been transfected with control (G–I, M) or MKP3 (J–L′, M) were prepared and cultured for 48 h with 4 nM Shh. The expression of Arx (G, K, red in G′, K′) and Nkx2.2 (H, L, red in H′, L′) and GFP (I, J, green in G′, H′, K′, L′) was examined by immunohistochemistry. The percentages of positive cells for Arx and Nkx2.2 in GFP-positive cells are shown in (M). (N–Q′) Treatment with chemical inhibitors for FGF signal blocks FP differentiation induced by Shh. (N) Schematic representation of the experiment. Explants were treated either with 4 nM Shh for 48 h (a), with 4 nM Shh and 500 nM of the MEK inhibitor PD184352 (b), or with 4 nM Shh and 1 µM of the FGF receptor inhibitor SSR128129E (c) for 12 h followed by incubation with 4 nM Shh for 36 h. The expression of Arx (O, P, Q) and Nkx2.2 (O′, P′, Q′) was assayed by immunohistochemistry. (R) Blockade of FGF/MAPK/ETV signaling pathway inhibits FP differentiation. Explants electroporated with FGFR1ΔC, MKP3, or EnR-ETV5^DBD^ were prepared and incubated with 4 nM Shh for 48 h, and expression of the FP marker Nato3 and the p3 marker Nkx2.2 were assayed by qRT-PCR. Relative expression levels were calculated by comparison to explants treated with control medium for 48 h.

We next asked whether FGF/MAPK activity is necessary for FP induction in vivo. First, we in ovo electroporated a dominant-negative FGFR1 in which the intracellular portion of the protein containing the kinase signaling domain is truncated [34]. We targeted the posterior region of HH stage 8 embryos, comprising the preneural tube and stem zone [29],[35]. Assaying embryos 48 hpt revealed Nkx2.2 expression in place of Arx (5/6; Figure 2B,B′,b1–b3). Transfection of a GFP control construct did not disrupt FP formation (0/10; Figure 2A,A′,a1–a3). Next, to inhibit FGF signaling downstream of the receptor, we transfected HH stage 8 embryos with MAP Kinase Phosphatase 3 (MKP3; also known as DUSP6) [36],[37], which dephosphorylates ERK1/2 and thereby inactivates the MAP kinase pathway. As a result, Arx expression was down-regulated and replaced by Nkx2.2 expression (4/6; Figure 2C,C′,c1–c3). Neither perturbation led to a significant change in total number of Arx, and Nkx2.2-expressing cells greatly changed, suggesting that changes were mainly due to the alteration in gene expression (Figure 2F). Together these data indicate that the FGF/ERK signaling pathway is required for the induction of FP in vivo.

We next assessed the spatial-temporal requirement for FGF signaling. At spinal levels of the chick central nervous system, MAP kinase is active in the caudal neural plate and stem zones [37]. Nevertheless, FGF receptors are expressed throughout the neural tube [37], and FGF signaling has been shown to play local roles even after the neural tube is closed [25],[38],[39]. To test whether the requirement for FGF signaling for FP differentiation was restricted to the regions of MAP kinase activity, we electroporated FGFR1ΔC or MKP3 into the neural tube and anterior preneural tube of HH stage 10- embryos, the region flanked by the posterior 4–5 somites. In contrast to the result of blocking FGF signaling in the preneural tube (Figure 2A–C′), Arx expression remained intact 48 hpt (6/7; 1/7 slightly down-regulated in the case of FGFR1ΔC and 0/6 for MKP3; Figure 2D,d1–d3,E,e1–e3). This result suggests that FGF signaling is required prior to prospective FP cells entering the neural tube.

We confirmed the requirement of FGF/ERK signaling for FP induction using ex vivo experiments. Explants were prepared from HH stage 9 embryos that had been in ovo electroporated with MKP3 [37] 3 h prior to the dissection and cultured for 48 h with 4 nM Shh. In these explants, FGF target genes *ETV5* [37],[40], *IL17RD/SEF* [41],[42], and *Nkx1.2* (see below) were more rapidly down-regulated than in control explants (Figure S3A,B). Compared to control explants (Figure 2G–I,M), MKP3 blocked the ability of Shh to induce Arx and increased the expression of Nkx2.2 (Figures 2J–L′,M and S3C–G′). A similar result was obtained when FGFR1ΔC was electroporated (Figure S3H–J,P). We also assayed [i] explants exposed to 4 nM Shh for 36 h that had been incubated in the presence or absence of the MEK inhibitor PD184352 [Figure 2N(b)] or the FGF Receptor inhibitor SSR128129E [43],[44] [Figure 2N(c)] for the first 12 h. Both treatments resulted in increased expression of Nkx2.2 (Figure 2O′,P′,Q′) at the expense of Arx (Figure 2O,P,Q). Together, this series of experiments indicate that neural progenitors require exposure to transient FGF/MAPK signaling at the time when Shh signaling is initiated in order to differentiate into FP.

**Figure 3:**
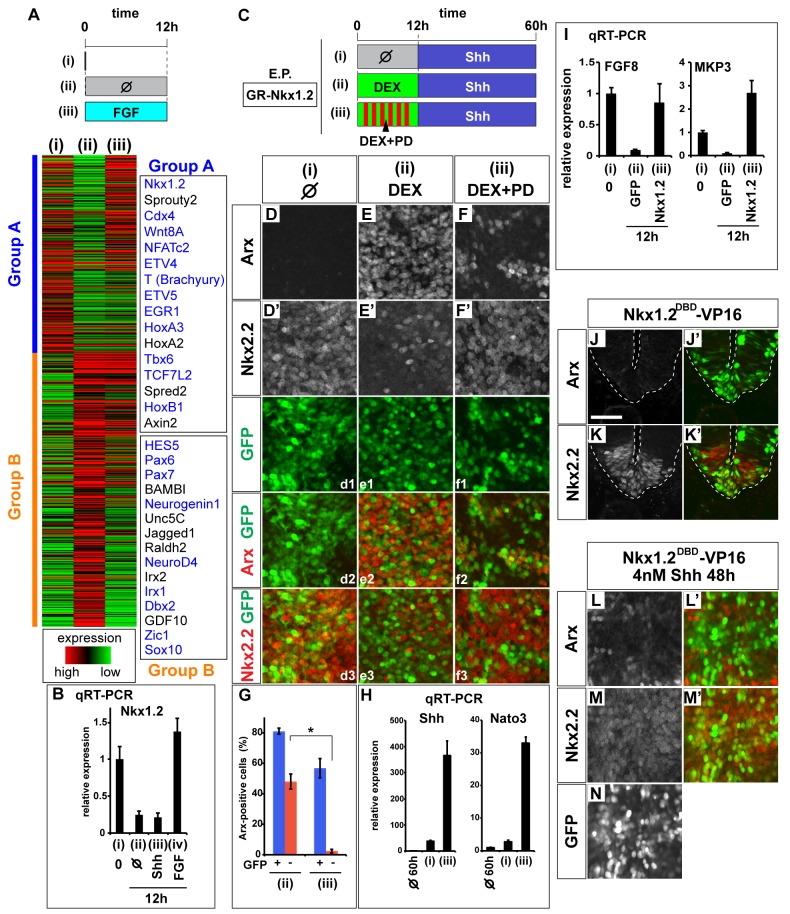
Nkx1.2 mediates FGF-dependent FP competence. (A) Histogram summarizing the mRNA-seq analysis of RNA from [i] explants taken immediately after preparation (i), after 12 h in vitro culture without (ii) or with 5 nM FGF (iii). The genes classified as Group A had higher expression levels in (i) and (iii) compared to (ii), whereas those in Group B had the opposite tendency. The transcription factors and signaling molecules tested in the secondary round of screening are shown in blue. The complete list of Group A and Group B is available in Table S2. (B) Nkx1.2 expression is regulated by FGF, but not by Shh. [i] explants were collected without incubation (i) or after a 12-h incubation (ii–iv) with the control medium (ii), with 4 nM Shh (iii), or with 5 nM FGF (iv), and the expression of Nkx1.2 was assayed by qRT-PCR. (C–G) Transient Nkx1.2 activity induces FP gene expression in a cell-autonomous manner. (C) Schematic representation of the experiment; [i] explants transfected pCIG-GR-Nkx1.2 were prepared and cultured in control medium for 12 h (i) or with 10 µM Dexamethazone [DEX; (ii)] or with DEX and 500 nM PD184352 [PD; (iii)] for 12 h followed by a culture with 4 nM Shh for 48 h. Explants were analyzed by immunohistochemistry for Arx (D, E, F, red in d2, e2, f2), Nkx2.2 (D′, E′, F′, red in d3, e3, f3), and GFP (green in d1–f3). (G) Quantification of Arx expression in GFP-positive and -negative cells. Most Arx expression was found in GFP-positive cells, and the frequency of Arx expression in GFP-negative cells was significantly decreased in condition (iii) (*p<0 .001; Student's *t* test). (H) The expression of FP genes by transient Nkx1.2 activity, determined by qRT-PCR. Explants incubated for 60 h without treatment or in the indicated conditions (C) and analyzed by qRT-PCR for the expression of Shh and Nato3. (I) Forced expression of Nkx1.2 in explants induces the expression of FGF8 and MKP3 after 12 h. Explants were harvested immediately after preparation (i), or after 12 h with control GFP (ii) or Nkx1.2 electroporation (iii), and analyzed by qRT-PCR. (J–N) Nkx1.2 is necessary for FP differentiation. (J–K′) Embryos were transfected with Nkx1.2^DBD^-VP16 expression vector at HH stage 8 and cultured for 48 h. The expression of Arx, Nkx2.2 (J, K, red in J′, K′, respectively), and GFP (green in J′, K′, L′) were analyzed by immunohistochemistry. Scale bar in (J) = 50 µm. (L–N) Nkx1.2 is necessary for FP differentiation in vitro. Explants transfected with Nkx1.2^DBD^-VP16 were incubated with 4 nM Shh for 48 h and analyzed by immunohistrochemistry for Arx and Nkx2.2 (L, M, red in L′, M′, respectively). Transfected cells were identified by GFP expression (N, green in L′, M′). In total, 8.73%±1.08% GFP-positive cells are Arx-positive (L), whereas 81.0%±1.09% are Nkx2.2-positive (M; see Figure 2M for control).

ETV4/5 (also known as Pea3 and Erm, respectively) are ETS family transcription factors expressed in neural cells competent to generate FP (see Figure 3A) and mediate transcriptional responses to FGF signaling [37],[40]. We therefore investigated the requirement for ETV4/5 activity in FP induction. In ovo electroporation with a dominant inhibitory version of ETV5 (EnR-ETV5^DBD^) at HH stage 8 [45] blocked Arx expression assayed 48 hpt (5/5) (Figure S3K–L′). In addition, we cultured explants electroporated with EnR-ETV5^DBD^ in the presence of 4 nM Shh for 48 h. As a result the induction of Arx was decreased and expression of Nkx2.2 increased (Figure S3M–P). We also examined the expression of another FP marker, Nato3, in explants electroporated either with MKP3, FGFR1ΔC, or EnR-ETV5^DBD^ by quantitative reverse transcription and polymerase chain reaction (qRT-PCR). Consistent with the findings from the immunohistochemistry of Arx, *Nato3* expression was significantly down-regulated compared to control explants and *Nkx2.2* expression up-regulated (Figure 2R). Together, these data indicate that FGF signaling through MAPK and ETV4/5 is necessary for FP development.

### Downstream Mediators of FGF Signaling in Neural Progenitors

To better understand the molecular mechanisms determining the competence for FP induction, we surveyed the transcriptome of FP-competent and -incompetent neural progenitors. For this purpose, we performed RNA-seq on samples extracted from competent explants harvested immediately after dissection (sample “0”) or explants treated with FGF for 12 h (“FGF_12 h”) and explants that had lost competence following incubation in vitro in control medium for 12 h (“0_12 h”) (Figure 3A).

From this analysis we selected 988 genes that displayed the greatest differences in their expression levels between explants assayed at the time of dissection [Figure 3A(i)] and explants cultured in vitro for 12 h [Figure 3A(ii)]. The genes expressed higher at 0 h than 12 h were categorized as Group A and further stratified using the expression level of genes in explants treated with FGF for 12 h [Figure 3A(iii)]. The genes that were expressed lower in 0 h than 12 h samples were categorized as Group B. Group A contained several genes related to FGF (including ETV4 and ETV5; Figure S3K–P), Wnt, and Eph/ephrin signaling as well as several transcription factors (Figure 3A, Table S2).

To test whether any of the Group A genes mimicked the activity of FGF signaling to prolong the competence period for FP induction, we prepared explants from embryos electroporated with a selection of these candidates (labeled blue in Figure 3A). Explants were incubated in control medium for 12 h and then exposed to Shh for an additional 48 h and assayed for Arx expression [see Figure 1E (ii)]. This secondary screen led us to focus our attention on the NK-type transcription factor Nkx1.2 (also known as Hox3, Sax1).

### Nkx1.2 Maintains FP Competence

Nkx1.2 is expressed in the caudal stem zone and preneural tube in chick and mouse embryos (Figure S4A) [46]–[48] and is induced by FGF signaling [24]. Shh signaling was not sufficient to maintain expression of Nkx1.2 in HH st10 [i] explants (Figure 3B) [24],[47],[48].

Hypothesizing that Nkx1.2 provides the FGF-dependent competence of cells to generate FP in response to Shh signaling, we devised a system in which Nkx1.2 expression could be manipulated to mimic the transient expression of Nkx1.2 during normal FP development. We prepared explants transfected with a construct encoding Nkx1.2 fused to the hormone-binding domain of Glucocorticoid Receptor (GR-Nkx1.2). In the absence of Nkx1.2 activation, no Arx expression was induced [Figure 3C(i),D,D′,d1,d2]. By contrast, transient activation of Nkx1.2 in explants by Dexamethasone treatment (DEX: a Glucocorticoid analogue) for the initial 12 h followed by an additional 48 h with Shh resulted in the induction of a substantial number of Arx-expressing cells [Figure 3C(ii),E,E′,e1,e2,G(ii)]. However, the presence of both GFP-positive/Arx-positive and GFP-negative/Arx-positive cells suggested that Arx has been induced non-cell-autonomously as well as cell-autonomously. A similar non-cell-autonomous induction of Arx was observed following the sustained expression of Nkx1.2 (Figure S4B–D′). This suggested that Nkx1.2 induced a secreted factor(s). We therefore performed qRT-PCR in explants transfected with Nkx1.2 and found that FGF8 and its target gene MKP3 were induced at 12 h after Nkx1.2 induction (Figure 3I). These findings suggest FGF8 and Nkx1.2 form a positive feedback loop and maintain the competence of cells to differentiate into FP.

To test whether FGF signaling is necessary for FP competence in cells expressing Nkx1.2, we repeated the 12 h activation of the Nkx1.2 experiment in the presence of the MAPK inhibitor PD184352. In this condition, Arx continued to be induced and these cells were derived from cells that had expressed Nkx1.2 [Figure 3C(iii),F,F′,f1,f2,G(iii)]. Other FP markers were also induced in this experimental regime [Figure 3C(iii)], as examined by qRT-PCR (Figure 3H). The expression of Nkx2.2 expression was reciprocal to Arx in these explants (Figure 3D′,d1,d3,E′,e1,e3,F′,f1,f3). These data indicate that transient expression of Nkx1.2 immediately followed by exposure to Shh is sufficient to reconstitute FP induction even when FGF signaling is blocked.

We next tested whether Nkx1.2 is required for FP differentiation. Nkx1.2 contains a Groucho-binding domain and appears to act as a transcriptional repressor [49],[50]. We therefore hypothesized that an activator variant—Nkx1.2^DBD^-VP16—would function as a dominant negative. Consistent with this, forced expression of Nkx1.2^DBD^-VP16 repressed Arx (7/8) and Nato3 (6/8) expression in vivo (Figure 3J,J′ and unpublished data) and promoted the ventral expansion of Nkx2.2 (7/8; Figure 3K,K′). In addition, in [i] explants expressing Nkx1.2^DBD^-VP16, Arx induction by Shh was blocked cell-autonomously and cells expressed Nkx2.2 instead (Figure 3L–N). Taken together, these findings suggest that Nkx1.2 and/or a closely related factor(s) is necessary for FP induction.

### Repression of RA Signaling, Pax6, and Irx3 Allows FP Differentiation

**Figure 4:**
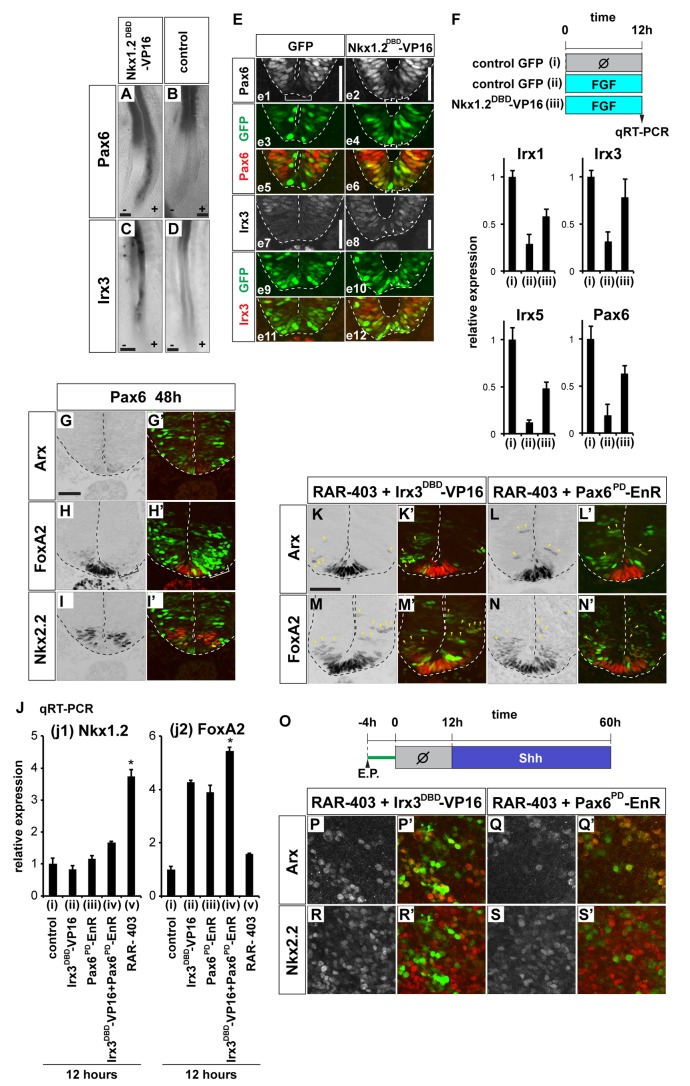
Nkx1.2 represses Irx3, Pax6, and RA activity to establish FP competence. (A–D) Irx3 and Pax6 are ectopically induced when Nkx1.2 activity is inhibited. Embryos were electroporated laterally with plasmids encoding a dominant-negative Nkx1.2^DBD^-VP16 (A, C) or control GFP (B, D) at HH stage 8+ and cultured for 12 h (for Pax6) or for 18 h (for Irx3). Whole-mount in situ hybridization was carried out for Pax6 (A, B) and Irx3 (C, D). In both cases, the right side (as viewed) of the embryo is electroporated. Scale bar (A, B, C, D) = 100 µm. (E, e1–e12) Irx3 and Pax6 are ectopically expressed in FP if Nkx1.2 function is inhibited. Either control (e1, e3, e5, e7, e9, e11) or Nkx1.2^DBD^-VP16 (e2, e4, e6, e8, e10, e12) were electroporated ventrally at HH stage 8+ and embryos cultured for 12 h. Expression of Pax6 (e1, e2, red in e5, e6) and Irx3 (e7, e8, red in e11, e12) were analyzed by immunohistochemistry. Transfected cells were identified by GFP expression (green in e3–e6, e9–e12). The aberrant expression of Pax6 and Irx3 in the FP is indicated by arrowheads (e2, e4, e6, e8, e10, e12). Scale bar (e1, e2, e7, e8) = 50 µm. (F) A dominant-negative Nkx1.2 aberrantly up-regulates intermediate neural genes. Explants electroporated with control (i, ii) or Nkx1.2^DBD^-VP16 (iii) were treated with control media (i) or with the medium containing 5 nM of FGF (ii, iii) for 12 h. The expression of the indicated genes was examined by qRT-PCR. (G–I′) Pax6 represses FP differentiation. HH stage 8 embryos were electroporated with the expression plasmid encoding Pax6 and incubated for 48 h. Immunohistochemistry for Arx, FoxA2, and Nkx2.2 (G, H, I, red in G′, H′, I′) indicated Pax6 represses Arx expression in the FP. The affected area is indicated by a bracket. Scale bar (G) = 50 µm. (J) A dominant-negative RAR (RAR-403) induces Nkx1.2, whereas the dominant-negative Pax6 (Pax6^PD^-EnR) and Irx3 (Irx3^DBD^-VP16) induce FoxA2. Explants electroporated with the indicated plasmids were incubated for 12 h and analyzed by qRT-PCR for Nkx1.2 and FoxA2 expression (*p<0.001; Student's *t* test). (K–S′) Inhibition of RAR and Irx3 or Pax6 activity, using RAR-403, Pax6^PD^-EnR, and Irx3^DBD^-VP16, prolongs the competence period of the neural explant cells to differentiate into FP. The indicated combinations of the dominant-negative expression plasmids were electroporated into the HH stage 8 embryos, and embryos were incubated for 48 h (K–N′) or explants (O–S′) were prepared and cultured with a delayed treatment of Shh as indicated in the schema (O). In (K–N′), ectopic expression of each gene is indicated by arrowheads. Scale bar (K) = 50 µm for (K–N′).

FGF signaling has been shown to inhibit the expression of several neural progenitor expressed transcription factors, including Pax6 and Irx3 [Figures S2R–T′ and S4K(iii)] and to perturb RA signaling (Figure S5A) [19],[21],[25]. The mRNA sequencing data were consistent with these findings (Group B in Figure 3A). We therefore asked whether Nkx1.2 activity was responsible for this repression. Electroporation of the dominant-negative Nkx1.2^DBD^-VP16 up-regulated expression of *Pax6* and *Irx3* (Figure 4A–F) without affecting FGF8 expression (Figure S5B–C′). This suggested that Nkx1.2 mediates the repressive activity of FGF signaling.

On the other hand, forced expression of either Irx3, Pax6, or a constitutive-active RA receptor (RAR-VP16) in HH stage 8+ embryos repressed FP differentiation (Figures 4G–I′ and S5D–I′) at 48 hpt. We therefore speculated that blocking the combined activity of these factors would prolong the competence of cells to generate FP. To test this, we electroporated, either alone or in combination, the dominant-negative versions of Irx3 (Figure S5J–K′), Pax6 [51], and RA Receptor RAR-403 [25]. Forced expression of individual constructs was not sufficient to induce FP (unpublished data). Electroporation of RAR-403 induced the expression of *Nkx1.2* [Figure 4J(j1)(v)], whereas the expression of *FoxA2* was induced by dominant-negative Irx3 and Pax6 (and their combination) [Figure 4J(j2)(ii)(iii)(iv)]. Strikingly, the combined expression of Irx3^DBD^-VP16 or Pax6^PD^-EnR with RAR-403 maintained the competence of cells to differentiate into FP both in vivo (6/8; Figure 4K–N′) and in vitro (Figure 4O–S′). Consistent with this, the FP markers Nato3 and FoxJ1 were also substantially induced when RAR-403 was transfected together with either Irx3^DBD^-VP16 or Pax6^PD^-EnR (Figure S5L). These results suggest that Nkx1.2 maintains FP competence by repressing the expression of transcription factors normally found in more mature neural progenitors.

To extend these findings, we investigated if FP differentiation also requires the blockade of RA signal in another species. For this, we took advantage of the differentiation of mouse ES cells into Arx-expressing FP cells. ES cells, cultured in serum free media, expressed FGF8 and Nkx1.2 (Figure S6E), and treatment with Shh for 60 h generated FP, as evidenced by the expression of Arx, *FoxA2*, and *Nato3* (Figure S6A,B,D). In contrast, the differentiation of FP was substantially reduced in ES cells exposed to RA in addition to Shh, and instead Nkx2.2-expressing p3 cells were generated (Figure S6A,C,D). In these assays, the addition of RA inhibited FGF8 and Nkx1.2 expression and up-regulated *Pax6* expression (Figure S6E). These findings are consistent with the idea that the blockade of RA is required for the FP differentiation in mouse as well as chick.

### Coincidence of Nkx1.2 and FoxA2 Induces FP

**Figure 5:**
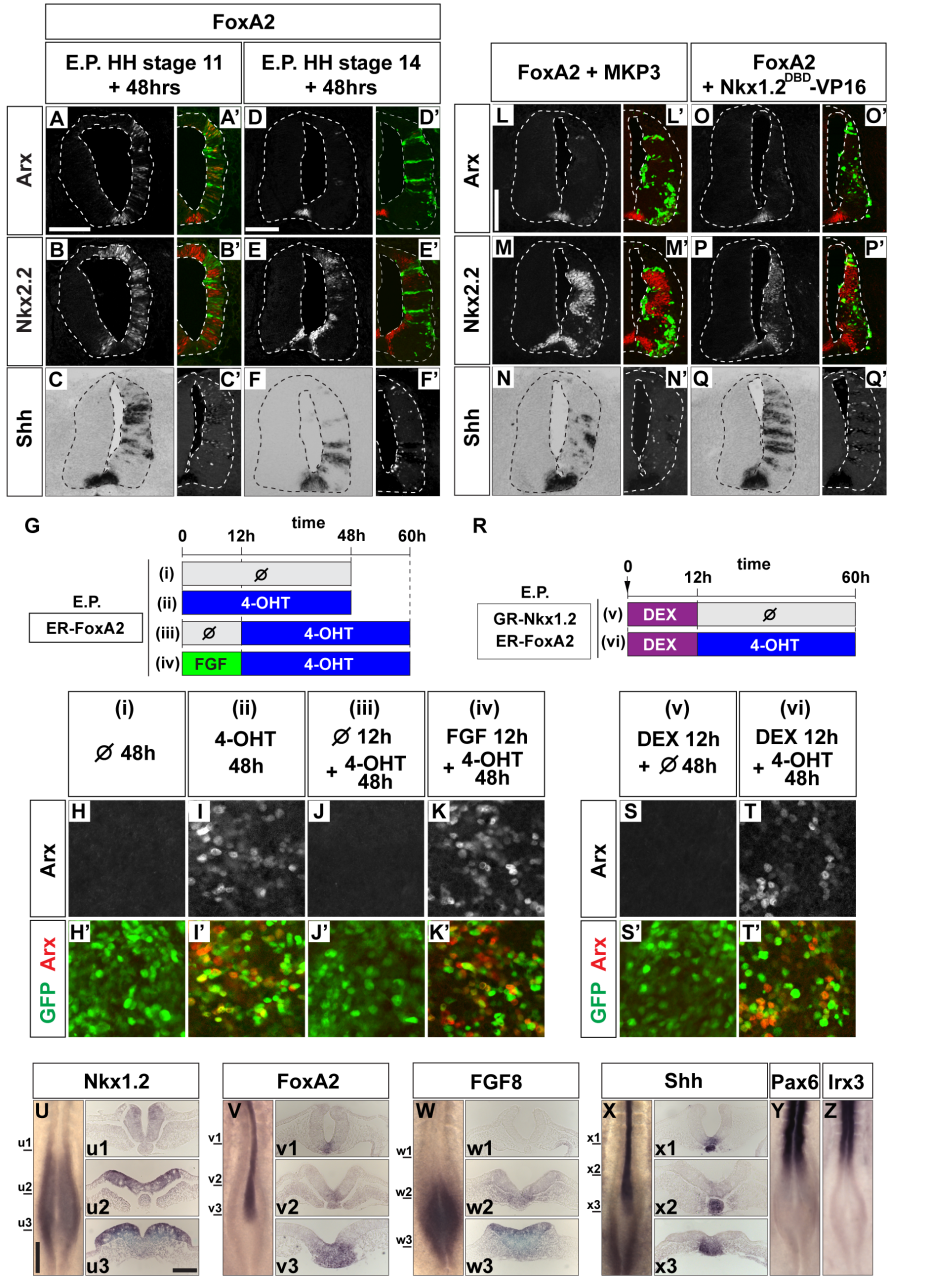
FP induction by FoxA2 requires FGF signaling. (A–F′) The ability of FoxA2 to induce Arx is time-dependent. Following the electroporation of a FoxA2 expression construct at HH stage 11 or at HH stage 14, embryos were cultured for 48 h. The expression of Arx (A, D red in A′, D′), Nkx2.2 (B, E, red in B′, E′), and Shh (C, F) were analyzed with immunohistochemistry (for Arx and Nkx2.2) or in situ hybridization (for Shh). Electroporated cells were identified by GFP (green in A′, B′, D′, E′, white in C′, F′). Scale bar (A for A, A′, B′, B′, C, C′ and D for D, D′, E, E′, F, F′) = 100 µm. (G–K′) Arx induction requires the early expression of FoxA2. ER-FoxA2 (the hormone-binding domain of the estrogen receptor fused to a full-length FoxA2 coding region) was electroporated into embryos. Explants were prepared and cultured as indicated in (G). 4-hydroxy-tamoxifen (4-OHT) was used at 1 µM. Explants were assayed for Arx (H, I, J, K, red in H′, I′, J′, K′) and GFP (green in H′, I′, J′, K′) expression. (L–Q′) The induction of Arx by FoxA2 requires FGF-derived factor(s). Expression plasmids for FoxA2 and MKP3 (L–N′) or Nkx1.2^DBD^-VP16 (O–Q′) were coelectroporated into the embryos at HH stage 11. Embryos were harvested at 48 hpt and analyzed for Arx (L, O, red in L′, O′), Nkx2.2 (M, P, red in M′, P′), and Shh (N, Q) expression. Electroporated cells were identified by GFP (green in L′, M′, O′, P′, white in N′, Q′). (R–T′) Sequential induction of Nkx1.2 and FoxA2 allows FP differentiation in vitro. GR-Nkx1.2 and ER-FoxA2 were electroporated and explants were prepared. These were cultured as indicated in (R). Dexthamethazone (DEX) was used at 10 µM. Arx (S, T, red in S′, T′) and GFP (green in S′, T′) expression was analyzed by immunohistochemistry. (U–Z) Comparison of the expression patterns of the indicated genes. HH stage 9+ embryos were analyzed by in situ hybridization with antisense RNA probes for Nkx1.2 (U, u1, u2, u3), FoxA2 (V, v1, v2, v3), FGF8 (W, w1, w2, w3), and Shh (X, x1, x2, x3), Pax6 (Y), and Irx3 (Z). The levels of the sections are indicated by lines in (U–X). Scale bars are in (U) = 200 µm (for U, V, W, X, Y, Z) and in (u3) = 100 µm (for u1–u3, v1–v3, x1–x3).

The Shh target gene FoxA2 is critical for FP induction [52]. Cells lacking FoxA2 fail to form FP (Figure S7A), and FoxA2 expression is initiated in prospective FP soon after neural induction and is maintained during FP differentiation [53] (Figure 5V). This prompted us to investigate whether the induction of FP by FoxA2 was also dependent on the competence of neural progenitors. Forced expression of FoxA2 in the posterior neural tube at an early time point (HH stage 11) induced Arx by 48 hpt, consistent with previous studies [10] (10/10; Figure 5A,A′). FoxA2 also induced its target gene *Shh*, and therefore Nkx2.2 was induced non-cell-autonomously (10/10 for each; Figures 5B–C′ and FS7G,G′ for negative control). By contrast, transfection of FoxA2 at a later time (HH stage 14) was not able to induce Arx (6/8;2/8 had sporadic expression; Figure 5D,D′), although ectopic expression of *Shh* and Nkx2.2 were still induced (7/8 each; Figure 5E–F′). Notably the induction of FP by FoxA2 was independent of Shh signaling, as the coelectroporation of PtcΔ, which inhibits the Shh signaling pathway [16], with FoxA2 did not abrogate the induction of ectopic FP (*n* = 8; Figure S7B–C″).

A requirement for early FoxA2 expression was also evident in explants. We constructed an inducible FoxA2 (an estrogen-receptor-fused FoxA2; ER-FoxA2; Figure 5G) and activated it for different time periods in explants prepared from transfected embryos. Induction of FoxA2 at the time explants were dissected and induced in Arx [Figures 5G(i)(ii),H–I′ and S7H). However, when the induction of FoxA2 was delayed for 12 h after dissection, it failed to induce FP [Figure 5G(iii),J,J′]. The ability of FoxA2 to induce FP after 12 h in culture could be restored by exposing the explants to FGF for the initial 12 h [Figures 5G(iv),K,K′ and S7H]. Consistent with this, blocking FGF signaling in vivo at HH stage 11, by transfecting MKP3, inhibited the ectopic induction of FP cells by FoxA2 (6/8 inhibited; Figure 5L,L′), although FoxA2 remained able to induce expression of *Shh* and Nkx2.2 (*n* = 6 for each; Figure 5M–N′). Thus, the timing and requirement for the competence of cells to induce FP in response to FoxA2 corresponds to the competence to induce FP in response to Shh.

We next asked if Nkx1.2 was involved in maintaining the competence of cells to induce FP in response to FoxA2. Forced expression of either Nkx1.2^DBD^-VP16, Pax6, or Irx3 with FoxA2 blocked induction of Arx, although ectopic *Shh* remained expressed, suggesting the FP induction by FoxA2 requires Nkx1.2 and the absence of Irx3 or Pax6 (more than five embryos out of six for each; Figures 5O–Q′ and S7D–F′ and unpublished data).

We sought to reconstitute FP induction in vitro by regulating the timing of Nkx1.2 and FoxA2 activity. To this end, we prepared explants from embryos coelectroporated with GR-Nkx1.2 and ER-FoxA2. Explants were treated with DEX for the first 12 h, to maintain Nkx1.2 activity, and then media was replaced with Tamoxifen, to induce FoxA2 [Figures 5R(vi),T–T′ and S7H]. This regime, but not conditions in which only GR-Nkx1.2 or ER-FoxA2 were activated [Figures 5G(iii),J,J′,R(v),S,S′ and S7H], resulted in the induction of Arx. Together these results suggest that the coincidence of FoxA2 and Nkx1.2 expression, which is determined by the intersection of Shh and FGF signaling, establishes the transcriptional code for FP induction.

Finally, in order to map where the expression of *Nkx1.2* and *FoxA2* intersect in vivo, we performed whole mount in situ hybridization. Whereas *Nkx1.2* was transiently expressed in the posterior stem and preneural tube (Figure 5U,u1–u3) [24],[46],[48], *FoxA2* expression was initiated just anterior to Hensen's node, and continued to be expressed in midline cells and notochord (Figure 5V,v1–v3). Therefore, the midline cells anterior to the Hensen's node (Figure 5u2,v2) appear to simultaneously express, albeit at low levels, *Nkx1.2* and *FoxA2*. In light of the ex vivo data (Figure 5K,K′,T,T′), it is highly likely that it is at this position cells acquire FP fate. Moreover the expression patterns of *FGF8* (Figure 5W,w1–w3) [37] and *Shh* (Figure 5X,x1–x3) [54] are consistent with those of *Nkx1.2* and *FoxA2*, respectively. By contrast, Pax6 and Irx3, which inhibit FP induction, are only expressed anterior to the limits of *FGF8* and *Nkx1.2* expression (Figure 5Y,Z). These in vivo observations support the idea (Figure 1E,H) that transient *FGF* and subsequent Shh signaling are critical for FP differentiation, whereas *Pax6* and *Irx3* restrict FP differentiation.

### FGF and Nkx1.2 Also Provide Competence for Neural Crest Induction

The induction of NCCs at the dorsal pole of the neural tube also depends on early exposure to inductive signals [20]. Consistent with this, ectopic BMP expression in the neural tube in vivo at early time points promoted the induction and delamination of NCCs [more than 7 embryos out of 10 for each, while Olig3 expression did not change significantly (*n* = 10); Figure S8A–C′]. By contrast, later exposure to BMP favored the generation of dorsal interneurons instead (*n* = 10; in all cases the expression of Sox10 and HNK1 were repressed while Olig3 expanded ventrally; Figure S8D–F′) [20]. This prompted us to address whether the switch in response to BMP dorsally is similar to the switch in response to Shh ventrally.

**Figure 6:**
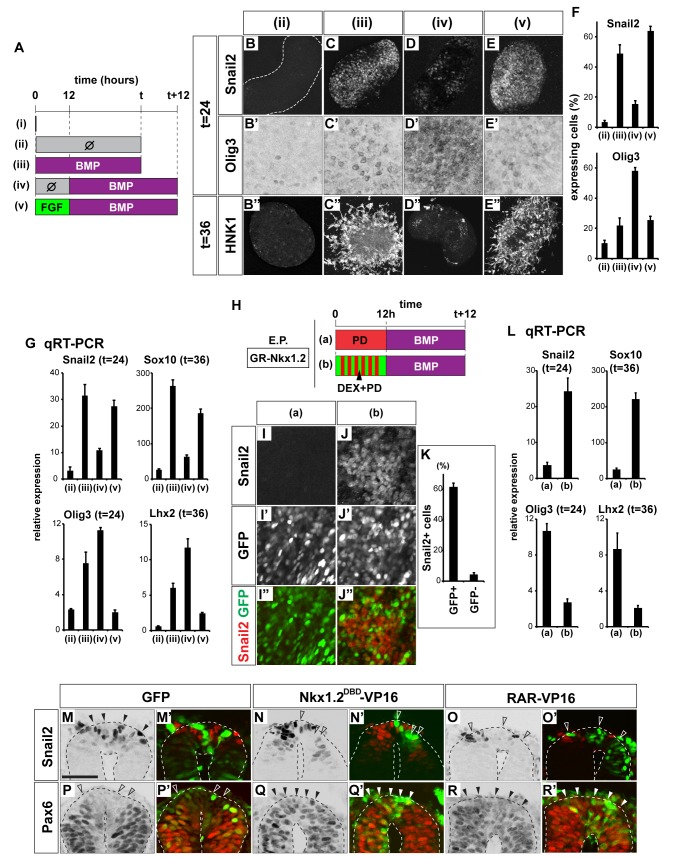
FGF signaling and Nkx1.2 expression provides competence for neural crest induction. (A–G) Neural crest induction requires early BMP signaling and the timing of competence is regulated by FGF signaling. Explants were cultured in the conditions indicated in (A) and analyzed by immunohistochemistry for Snail2 (B, C, D, E), Olig3 (B′, C′, D′, E′), and HNK1 (B″, C″, D″, E″). Note that the Olig3 staining, which gives a weaker signal, is shown at a higher magnification (B′, C′, D′, E′; 125 µm per side) than the other images (B, B″, C, C″, D, D″, E, E″; 375 µm per side). Quantification is shown in (F). (G) qRT-PCR was used to assay the expression of Snail2, Sox10, Olig3, and Lhx2 in explants cultured as indicated by the schema in (A). The relative expression levels compared to condition (i) are shown. (H–L) Transient expression of Nkx1.2 provides competence for the neural crest induction. Explants electroporated with GR-Nkx1.2 were cultured in the conditions described in (H). Explants indicated (PD) were treated with 500 nM PD184352 and all explants assayed by immunohistochemistry for Snail2 (I, J, red in I″, J″) and GFP (green in I″, J″). Quantification of (J–J″) provided in (K) suggests that the majority of Snail2-expressing cells derive from cells electroporated with GR-Nkx1.2. (L) qRT-PCR for expression of Snail2, Sox10, Olig3, and Lhx2. Relative expression levels compared to levels at time 0 were calculated. (M–R′) Inhibiting Nkx1.2 or enhancing of RAR-VP16 activity blocks neural crest induction. Control GFP (M, M′, P, P′), Nkx1.2^DBD^-VP16 (N, N′, Q, Q′), or RAR-VP16 (O, O′, Q, Q′) was electroporated at HH stage 8+ and embryos cultured for 12 h to reach HH stage 12. Assaying Snail2 (M, N, O, red in M′, N′, O′) and Pax6 (P, Q, R, red in P′, Q′, R′) expression indicated an inhibition of Snail2 expression in the dorsal midline of the neural tube and a dorsal expansion of Pax6. Affected cells are labeled with arrowheads. Scale bar in (M) for (M–R′) = 50 µm.

NCCs express Snail2, Sox10, and HNK1, whereas Olig3 is expressed in dorsal neural progenitors that generate dI1–dI3 interneurons [12],[13],[55],[56]. Using these as markers, we assayed the generation of NCCs in vitro. Treatment of [i] explants with 0.25 nM BMP4 resulted in the induction of the Snail2 [Figure 6A(ii)(iii),B,C,F,G] at 24 h and HNK1 and Sox10 at 36 h [Figure 6A(ii)(iii),B″,C″,G]. Migratory cells were also apparent by 36 h. However, if BMP treatment was delayed for 12 h after [i] explants were placed in culture, the induction of Snail2 was lost [Figure 6A(iv),D,G]. This did not appear to be due to the loss of responsiveness to BMP, as the induction of the dorsal neural progenitor marker Olig3 (dP1–dP3) and the dorsal interneuron dI1 marker Lhx2 was maintained in these conditions [20] (Figures 6B′,C′,D′,F,G and S8G–I). Moreover, explants treated at early and late times generated comparable levels of signaling activity, assayed using a luciferase reporter with a BMP-responsive element (Figure S8K) [18]. Strikingly, NCC induction was restored if explants were exposed to bFGF for 12 h prior to the treatment with BMP4 [Figures 6A(v),E–E″,F,G and S8J]. Thus these data suggest that the competence to induce neural crest differentiation is determined by FGF signaling.

Next we asked if the activity of FGF in the dorsal neural tube is also mediated by Nkx1.2. Overexpression of Nkx1.2 on its own did not induce any gene expression characteristic of the neural crest [Figure S8L(iii)], suggesting the cells still require BMP signal for neural crest induction. To test for a direct effect of Nkx1.2 on the neural crest induction, we transiently expressed Nkx1.2 and blocked FGF signaling simultaneously [Figure 3C(iii)]. We prepared [i] explants that had been electroporated with GR-Nkx1.2 and cultured these in the presence of PD184352 alone [Figure 6H(a)] or together with DEX [Figure 6H(b)] for 12 h. Media was then replaced with 0.25 nM BMP for an additional 24-h culture. Assaying NCC and dorsal neural progenitor markers revealed that the expression of Nkx1.2 promoted NCC induction [Figure 6H(b),J–L] and blocked dI1–3 generation (Figure 6L).

Finally we asked if Nkx1.2 is necessary for the neural crest induction by expressing the dominant-negative Nkx1.2^DBD^-VP16. This resulted in the cell-autonomous repression of Snail2 expression in vivo (4/6; Figure 6M–N′) and enhanced Pax6 expression (5/6; Figure 6P–Q′). Moreover, the overexpression of RAR-VP16 in the dorsal area inhibited the Snail2 induction at the expense of that of Pax6 (6/6; Figure 6O,O′,R,R′). The electroporation of Pax6 and Irx3 also inhibited Snail2 expression (5/7 for Pax6, 5/6 for Irx3; Figure S8M,M′ and unpublished data). Together these findings suggest that the competence of neural progenitors to generate NCCs is determined by the FGF-mediated expression of Nkx1.2, via repressing the activities of RA, Pax6, and Irx3.

## Discussion

**Figure 7:**
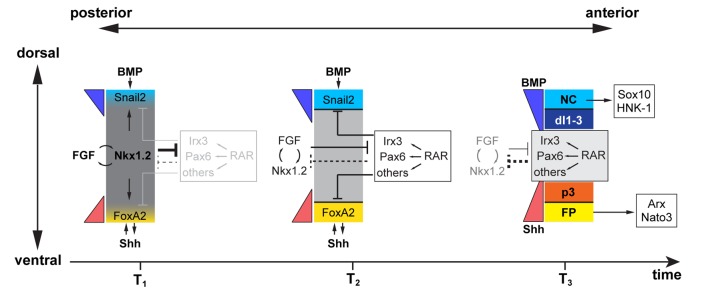
A model for the specification of FP and NCC. Cells in the posterior open neural plate area (pre-neural tube) (time T_1_) are exposed to FGF (drawn as a grey band). FGF and Nkx1.2 form a positive feedback loop and RAR/Irx3/Pax6 activity is low. Shh (red) and BMP (light blue) signal to progenitors at the poles of the forming neural plate. As a consequence of axis elongation, progenitors are displaced anteriorly into the neural tube, FGF signal decreases (time T_2_), and Nkx1.2 is down-regulated. Cells are no longer competent to induce FP or NCC. The combination of RAR/Irx3/Pax6 inhibits Nkx1.2 expression, either directly or indirectly (as indicated by the dashed line), and provides the competence for Shh and BMP signaling to induce the neuronal progenitors (time T_3_).

In this study we describe a molecular mechanism that controls the spatial-temporal competence of neural progenitors (Figure 7). Previous studies have revealed that the specification of NCC and FP depend on developmentally earlier inductive signaling than the progenitors of neuronal subtypes [10],[20]. Our data provide evidence that this timing is set by FGF signaling. Emanating from the posterior pole of the neural tube, FGF induces the expression of the NK-1 homeodomain factor Nkx1.2 to establish a region of FP and NCC competence. The range of FGF signaling confines this competence region to the caudal preneural tube [24],[35],[37]. Within these cells, Shh, produced from the underlying mesoderm, initiates FP induction by activating FoxA2, and BMP signaling dorsally initiates NCC induction. At this position in the embryo, the extent of Shh and BMP signaling restricts induction of FP and NCC to the prospective ventral and dorsal poles of the neural tube, respectively. As development proceeds, axis elongation results in the posterior regression of FGF signaling, thereby restricting Nkx1.2 expression and FP/NCC competence to the caudal region of the embryo. More mature neural progenitors, which have exited the region of FGF signaling, lack Nkx1.2 expression and generate distinct classes of neuronal progenitors in response to the increasing amplitudes of Shh and BMP signaling. Together, these data reveal how the cell movements responsible for axial elongation are exploited to change the signals to which progenitors are exposed and thereby impose shifts in the transcriptional program of cells that alter the competence of prospective neural cells to inductive signals.

### Transient FGF Signaling Provides FP and NCC Competence

Classic embryological grafting studies provided the first evidence that a signal, later identified as Shh, produced by the notochord is responsible for FP induction [57]–[60]. These studies also found that the capacity for FP induction attenuated as neural cells matured. This loss of competence restricts the specification of FP to the ventral midline of the neural tube by limiting the homeogenetic induction of FP [57]. Our study reveals a molecular mechanism that explains these observations. The intersection of FGF and Shh signaling is restricted to regions of the neural plate immediately anterior to the regressing node (Figure 5W,X). This function for FGF signaling complements its previously identified role as an inhibitor of neuronal differentiation in this region of the embryo [21],[48]. In these regions the low levels of Shh emanating from axial mesodermal cells mean that the only Nkx1.2-expressing cells that receive sufficient Shh to induce FoxA2 are those in the ventral midline [10],[61].

The function of FGF and Shh signaling in FP induction is also supported by data from the directed differentiation of ES cells to dopaminergic neurons [62],[63]. This cell type is generated by FoxA2-expressing progenitors at the ventral midline of the midbrain, and their in vitro differentiation requires FGF signaling transiently during the period ES cells commit to a neural fate [63],[64].

The coincidence of FGF and BMP signaling is required for NCC specification. This is in good agreement with studies that have implicated FGF signaling in the specification of NCC (Pax7, Zic1, and Msx1 expression) as early as gastrula stages [65],[66] and the subsequent determination of neural crest fate by BMP and other signals (e.g., Wnt, Notch) [67]. The functional difference between FGF, Wnt, and Notch in NCC specification remains unclear. Nevertheless, our data implicate a regulatory network at least between FGF and Wnt because FGF induces Nkx1.2 expression that in turn induces Wnt gene expression (Figure S4K). Taken together, therefore, in both the dorsal and ventral neural tube, the intersection of anterior–posterior FGF signaling with dorsal–ventral morphogen signaling provides a spatial and developmental time window that determines the induction of the cell types characteristic of the poles of the neural tube.

By contrast, a study from chick embryos suggested that ectopic expression of FGF inhibits NCC specification and emigration [56]. The lack of NCC induction in these experiments could be due to the prolonged activation of FGF signaling because our studies indicate that sustained FGF exposure inhibits NCC production (Figure S8N–P′). In this view, therefore, FGF signaling is transiently required to establish NCC competence, but its sustained activity blocks the elaboration of NCC identity [56]. FP induction also displays a similar requirement for transient FGF signaling (Figures 1K–N′ and S2J,K). In vivo the transience of FGF signaling is determined by the posterior regression of the source of FGF driven by axis elongation [68]. The consequent down-regulation of FGF signaling in neural tissue as it becomes incorporated into the neural tube therefore allows the elaboration of FP and NCC identity that are specified earlier. This mechanism exploits tissue morphogenesis to coordinate progression in cell identity with the overall dynamics of the embryo's development.

Loss of FGF signaling also prompts the down-regulation of Nkx1.2 expression and a change in the competence of progenitors not committed to FP or NCC identity. These cells now respond to the dorsal and ventral morphogens by acquiring identities of neuronal progenitors. In the ventral neural tube, the increasing levels of Shh production induce p3 identity in the cells dorsal to the FP and MN progenitors at a further distance [17]. Dorsally progenitors of dI1–3 neurons are induced by BMP signaling [20]. Taken together these data reveal how the cell movements that drive axis elongation provide a timing mechanism for changes in competence by controlling the combination of signals to which cells are exposed. This increases the diversity of the cell types generated in the neural tube and ensures their correct temporal and spatial generation.

### Nkx1.2 Establishes the Competence for the FP and NCC Differentiation

The competence to form FP and p3 progenitors appears to be mutually exclusive, as does the formation of NCC and dorsal interneurons. FGF has been shown to block both the induction of Shh-dependent neuronal subtypes in the ventral neural tube and the expression of transcription factors that define the progenitors of these neurons [21],[25]. This does not appear to be a consequence of substantial changes in Shh signal transduction (Figure S2I). Likewise BMP signaling in neural progenitors appears unaffected by FGF signaling (Figure S8K). Moreover the induction of FP and NCC identity within cells receiving FGF signaling suggests that there is not a complete blockade in the specification of new cell identities. Instead, FGF signaling appears to act by regulating the expression of a set of transcription factors in neural progenitors that transform the transcriptional program induced by Shh or BMP.

Our attention focused on Nkx1.2, as this appeared to mediate the FGF-dependent competence for FP and NCC differentiation. In support of this, Xenopus Nkx1.2 gene (Nbx) [69] is expressed in the presumptive neural crest area and is essential for the neural crest differentiation. Nevertheless, mutation of Nkx1.2 in mouse embryos does not appear to affect FP or NCC generation [70]. Redundancy with Nkx1.1, an Nkx1.2 paralogue, may explain this apparent discrepancy. Both Nkx1.1 and Nkx1.2 are expressed in similar regions of the caudal embryo and forced expression of Nkx1.1 had similar effects to Nkx1.2 (Figure 5U and unpublished data). The generation of compound mutant mice lacking both genes would test this hypothesis. Alternatively there might be functional redundancy among a broader set of transcription factors in the caudal preneural tube, and it will be important to understand the function of these and the transcriptional network that connects them.

A recent study has identified changes in higher order chromatin structure of specific genes as cells progress from the pre-eural tube to the neural tube [71]. How these changes are instated remains to be determined. It is possible Nkx1.2 regulates chromatin modifiers or factors that direct the chromatin modifiers to appropriate regions of the genome. Alternatively other targets of FGF signaling, independent of Nkx1.2, could be responsible. Irrespective of the mechanism, the irreversible changes in chromatin structure might provide an explanation as to why cells that have lost their competence to differentiate into the FP do not regain it even if exposed to FGF. In this context, it is notable that in pancreatic development, the repression of Arx expression is controlled by methylation of a CpG island within the Arx gene locus [72]. This mechanism does not seem directly applicable in the neural tube, however, because neural explants treated with 5-aza-dC, a DNA demethylating agent, did not alter the expression of key patterning genes (unpublished data). Identification and detailed analysis of the regulatory regions and epigenetic marks will be necessary to explore the relevant mechanism further.

### A Gene Regulatory Network for FP and NCC Competence

Expression of Nkx1.2 repressed expression of neural progenitor transcription factors, including Pax6 and Irx3 (Figure S4E–K). Conversely, our experiments and previously published studies indicate that Pax6, Irx3, and RA signaling inhibit FP and/or NCC differentiation [19],[21],[73], while promoting the establishment of neuronal progenitor identity. Although nonautonomous effects of Nkx1.2 might contribute, cell-autonomous mutual cross-repression between alternative transcription states is a reoccurring theme in developmental decisions and appears to be the most likely explanation for the spatial and temporal transition between the different competence states. Indeed cross-repressive interactions are apparent between the transcription factors that determine distinct progenitor domains along the dorsal–ventral axis of the neural tube [74]. Thus there appears to be a common logic that underlies the transcriptional mechanisms along both the dorsal–ventral and rostral–caudal axis of the neural tube.

It is notable that as well as being induced by FGF signaling, Nkx1.2 also promotes the expression of FGF (Figure 3I). This establishes a positive feedback loop that supports the FP/NCC competence state. This is reminiscent of the positive feedback loop between FoxA2 and Shh expression that is characteristic of the FP itself. In both cases the feedback loop functions to repress the expression of Pax6/Irx3 in a cell-autonomous manner (Figure S4L–O″) and must be interrupted in order to limit the homeogenetic induction of FP cells [57],[58]. In the case of the FoxA2-Shh loop, the change in progenitor competence mediated by the down-regulation of FGF signaling is responsible for ending the feedback loop. In the case of the FGF-Nkx1.2 loop, it seems likely that RA signaling terminates the positive feedback. RA emanating from somites adjacent to the maturing neural tube forms a rostral to caudal gradient in both neural tissue and paraxial mesoderm that counteracts the posteriorly produced FGF [21]. Consistent with this, a dominant-negative RAR effector is sufficient to induce Nkx1.2 expression in cells that would otherwise have down-regulated its expression [Figure 4J(v)]. Thus axis elongation not only results in the posterior regression of the source of FGF but also the exposure of cells to RA signaling [21]. Once RA starts to be produced in the somites, the inductive effect on Pax6/Irx3 by RA overcomes the repressive effect of Nkx1.2 on their expression and cells change the competence in response to Shh or BMP, and this promotes the transition in competence, adding a further level of spatial and temporal control over the transition (Figure 7).

The details of the transcriptional network controlled by Nkx1.2 that acts to induce FP or NCC fate in response to Shh or BMP signaling remains to be fully elucidated. The set of transcription factors responsible for defining premigratory NCC identity is known in some detail, and it will be interesting to examine how Nkx1.2 influences this network [75]. For FP differentiation the situation is not as clear. Our data suggest that FP is specified within 12 h of the initiation of FGF and Shh signaling. Nevertheless, it takes more than 30 h before expression of mature FP genes such as Arx, Nato3, and HES1 (Figure 1J and unpublished data). We anticipate that transcriptional mechanisms must relay the immediate targets of FGF signaling (Nkx1.2) and Shh signaling (FoxA2) to regulate the mature FP genes.

Taken together, the current study provides new insight into how the interplay between cellular competence and inductive signals controls pattern formation and increases cell type diversity in the neural tube. A striking feature of this mechanism is that it combines the morphogenetic movements of the developing embryo with signals acting along orthogonal axes to position and time transitions in competence. The dynamics of these interactions offer a means to couple changes in the response of individual cells to the overall development of the embryo. More generally, the increasingly detailed knowledge of the gene regulatory network underpinning these events makes the neural tube a good model in which long established developmental concepts, such as competence and inductive signals, can be understood in mechanistic terms.

## Materials and Methods

### Genes

The Ensembl database (http://www.ensembl.org/index.html) and National Center for Biotechnology Information (NCBI; http://www.ncbi.nlm.nih.gov) accession numbers are as follows: chicken Arx (ENSGALG00000025770), chicken Nkx6.1 (AF102991), chicken Olig2 (AF411041), and chicken FoxP2 (ENSGALG00000009424). Refer to Table S1 for the other genes.

### In Ovo Chick Electroporation, Immunohistochemistry, and in Situ Hybridisation

All animal experiments were performed under a UK Home Office project license within the conditions of the Animals (Scientific Procedures) Act 1986. All authors are personal license holders, and this study was performed under the project license PPL80/2528, approved by the Animal Welfare and Ethical Review Panel of the MRC-National Institute for Medical Research.

Unless otherwise stated, in ovo electroporation experiments were performed using the pCIG expression plasmid, which contains an IRES-GFP gene downstream of the gene of interest [76]. For the overexpression experiments of Shh, pCX-Shh-N (an expression construct producing the amino-terminal region of Shh) was used [77]. Electroporation was performed at the indicated stages using an ECM-830 electroporator (BTX). For the early stage electroporations, DNA was applied with a glass capillary onto the open neural plate and electric pulses given from dorsal to ventral. Otherwise the DNA was placed in the lumen of the neural tube and electroporation was performed laterally. At the indicated time points, embryos were fixed with 4% paraformaldehyde (PFA), subsequently treated with 15% of sucrose, embedded in gelatin, and 14 µm sections taken. The antibodies used in this study were against Arx (rabbit, a gift from J. Chelly) [78], Irx3 (rabbit, a gift from T.M. Jessell) [25], Nkx2.2 (mouse, DSHB 74.5A5), Olig2 (rabbit, Millipore AB9610) and Pax6 (rabbit, Millipore AB2237), Olig3 (mouse, Abcam ab168573; rabbit, SIGMA HPA018303), Sox10 (rabbit; Abcam ab27655), Snail2 (rabbit; Cell Signaling Technology 9585), HNK1 (mouse; BD 347390), GFP (sheep, Biogenesis 4745-1051; rabbit, Invitrogen A11120), haemagglutinin (rabbit, SIGMA H6908), FoxA2 (goat, Santa Cruz sc-6554X), FoxP2 (rabbit, Abcam ab16046), and Nato3 (rat, a gift from Y. Ono) [27]. For in situ hybridization of chick and mouse embryos, embryos were harvested at the indicated stages and fixed with 4% paraformaldehyde (PFA) overnight. Antisense RNA probes were synthesized with Digoxigenin (DIG; Roche), and hybridization was performed at 70°C in the solution containing 1.3×SSC (saline-sodium citrate) pH 5.0, 5 mM EDTA, 1 mg/ml torula yeast RNA (SIGMA), 0.2% Tween 20 detergent (SIGMA), 0.5% CHAPS detergent (SIGMA), 100 µg/ml Heparin sodium salt, and 50% Formamide. Signals were developed by BM-Purple (Roche). In situ hybridization on chick sections was performed as described previously [79].

### Transient Expression System

The hormone-binding domain of the estrogen receptor (ER) was fused to Gal4-VP16 to make pCIG-ER-Gal4-VP16 [32]. The target gene HA-MKK1SD (a haemagglutinin-tagged constitutive-active MKK1; [33]) was placed under the control of 14 concatemerized Gal4 binding sequences (the upstream activating sequences; UAS). For transient in ovo expression, pCIG-ER-Gal4-VP16 and 14×UAS-HA-MKK1SD were electroporated at HH stage 8. For the induction of the target gene, Pluronic gel F-127 (SIGMA) was prepared to 20% (w/v) in Hank's Balanced Salt Solution (HBSS; SIGMA), and 4-Hydroxytamoxifen (4-OHT; SIGMA) was added to 50 µM. The gel was placed between the vitelline membrane and the embryo. After 8 h the gel was washed out with HBSS and the embryos were incubated until the indicated time point. For the sustained expression, the gel was replenished every 12 h. pCIG-ER-FoxA2 was generated by fusing the same ER domain to the coding region of mouse FoxA2 gene, and pCIG-GR-Nkx1.2 was generated by fusing the hormone-binding domain of human Glucocorticoid Receptor (also known as NR3C1) [80] to the coding region of mouse Nkx1.2 gene.

### Explants

Explant assays were performed as described previously [81],[82]. Briefly, explants were prepared from the posterior neural epithelial layer of HH stage 9+ embryos and maintained in Leibovitz (L-15) medium (Gibco) during the preparation, embedded in a drop of collagen (SIGMA) buffered with DMEM (SIGMA), and preincubation was performed under 5% CO_2_ and 37°C for 1 h to allow the collagen drop to harden. Explants were then cultured with F-12/Ham (Gibco) containing Glutamine supplemented with antibiotics (50 U/ml of Penicillin, 50 µg/ml of Streptomycin) and Mitoserum (BD). Mouse recombinant FGF2 was purchased from R&D, and mouse Shh (C25II) [54],[83] was produced in house. In addition, in Figure 1E(iii), we tried DKK (R&D) to inhibit Wnt, BMS 493 [84], and 4-Diethylaminobenzaldehyde (DEAB) [85] to inhibit RA and valproate as a Notch activator [86]. In Figure 2N–Q′, FGF signal was inhibited by PD184352 (SIGMA) or SSR128129E (Selleckchem) [43],[44]. In these experiments, explants were prepared in L-15 in the presence of either of the inhibitors. Inhibitors were also added to the collagen. Explants were fixed with 4% PFA for 1 h and stained with antibodies as indicated. Data were collected with an SP2 confocal microscope (Leica), and all explant images are 125 µm per side, except in Figure 6B,B″,C,C″,D,D″,E,E″, which are 375 µm. Quantitation was performed on at least three areas, each of which contained approximately 200–250 cells, randomly chosen from the explants. Data are presented as mean values ± s.e.m.

### Reporter Assays

The reporter constructs used in this study were as follows: the GBS-Luc reporter construct (the Firefly Luciferase gene driven by 8 concatermized Gli-binding sites) [87], the 14×UAS-luc (the luciferase gene controlled by 14×UAS sequences), the RARE-luc (a construct with a triple repeat of the RA Responding Element; obtained from Addgene) [88], and the BRE-luc (a gift from P. Ten Dijke) [18]. In ovo reporter assays were performed by co-electroporating the reporter together with pRL-CMV (Promega; used as the normalization control). The anterior thoracic levels of the embryos were harvested at indicated time points, and luciferase assays were performed following the manufacturer's instruction (Dual luciferase assay kit; Promega) using a Luminometer 9509 (Berthold). The relative induction levels of the luciferase were calculated by comparison with control electropotation. At least five embryos were assayed at each condition and were represented as mean values ± s.e.m. For the reporter assays in explants, five explants were pooled for each measurement, and four pools were assayed for each condition. Relative induction levels were calculated compared to the luciferase activity in unstimulated control explants.

### qRT-PCR

RNA was extracted from a pool of 20–30 explants using Picopure RNA extraction kit (LifeTechnologies) to obtain 500 ng–1 µg of total RNA. cDNAs were synthesized by Superscript II reverse transcriptase (Invitrogen), and qRT-PCR analyses were performed by 7900 HT Fast Real-Time PCR system (Applied Biosystems). Where possible, the primers were designed to cross an intron by referring to Ensembl database in order to avoid amplifying any contaminating genomic DNA. Sequences of primers are presented in Table S1. Each gene expression level was normalized to that of β-actin. Each data point contains at least two biological replicates and is presented as the mean values ± s.e.m.

### Expression Plasmids

Nkx1.2^DBD^-VP16 and Nkx1.2^DBD^-EnR were made by fusing the transcription activator domain of the herpes simplex virus VP16 or drosophila Engrailed repressor domain and the DNA-binding domain of mouse Nkx1.2 (amino acid numbers 131–218). Likewise, the DNA binding domain of mouse Irx3 (amino acid numbers 131–200) and mouse ETV5 (amino acids numbers 290–489) [45] were used for Irx3^DBD^-VP16, Irx3^DBD^-EnR, and EnR-ETV5^DBD^. A nuclear localization signal was added to Irx3 and ETV5 constructs. The construction of FGFR1ΔC [34], Pax6^PD^-EnR [51], RAR-VP16, RAR-403 [25], FoxA2-EnR [10], and PtcΔ [16] were described previously.

### High Throughput mRNA Sequencing Analysis

Libraries were synthesized with TruSeq™ RNA Sample Preparation kit according to the manufacturer's instruction (Illumina). Fifty-five base-pairs paired-end sequencing was performed on a HiSeq 2000 (Illumina). From 50 to 100 million clusters were obtained from each sample. The sequence data were mapped using Bowtie [89] to the 16,832 chick cDNAs annotated in the Ensembl database. Sequences mapped to each gene were then counted and each sample normalized to the total number of reads in the sample. The standard deviation of each gene across the samples was calculated, and the 988 genes that had the standard deviations of more than 0.5 were selected. These were divided into two groups: those with higher expression at time 0 than that in 0 nM_12 h (416 genes) and a second group with lower expression at time 0 than in 0 nM_12 h (572 genes). The genes that restored by FGF (263 genes; Group A) and the genes repressed by FGF (364 genes; Group B) by up to 10-fold were selected for further study. The list of the categorized genes is available in Table S2. The original sequence data have been deposited in ArrayExpress (http://www.ebi.ac.uk/arrayexpress; E-MTAB-2393).

### Mouse Embryonic Stem (ES) Cells

Mouse ES cells were maintained on feeders in LIF-supplemented medium. Cells were differentiated using a monolayer differentiation protocol with minor modifications [90]. Briefly, feeder-depleted ES cells were plated at high density on gelatin-coated CellBind dishes (Corning) and maintained in N2B27 medium. For FP differentiation, recombinant 2 µg/ml Shh was added to the medium at day 3.5 and cells were cultured for an additional 60 h. For p3 differentiation, 30 nM RA (SIGMA) was added at day 3 and replaced each day with media containing 30 nM RA and 2 µg/ml Shh for an additional 60 h. In qRT-PCR, the expression values of each gene were normalized to that of RhoA, which is expressed at the same level throughout the differentiation.

## Supporting Information

Figure S1 Transfected Shh affects the contralateral side of the neural tube. (A) Schematic representation of the experiment. pCX-Shh-N or control GFP was electroporated at HH stage 8, and embryos were cultured for 12 h. Explants were embedded in collagen drops and incubated for 1 h so that the collagen could harden. The explants were then incubated in the medium for an additional 36 h. (B–E″) Overexpressing Shh-N, but not GFP, ventralizes the intermediate neural explants not only on the ipsilateral but also on the contralateral side. Explants were analyzed by immunostaining for GFP (B, C, D, E), Arx (B′, C′, D′, E′), and Nkx2.2 (B″, C″, D″, E″) expression. Arx (C′) and Nkx2.2 (D′) expression was found in GFP-negative explants, suggesting that Shh-N spread contralaterally within 12 h of electroporation. DAPI staining in (E″′).
(TIF)

Figure S2 Expression of ventral neural genes and a regulatable expression system for the chick neural tube. (A–F) Expression pattern of Arx (A, C, E), Nkx2.2 (B, C, F), and FoxA2 (D, E, F) at HH stage 24. Arx and Nkx2.2 expression are mutually exclusive (C), whereas FoxA2 is expressed in the FP and the p3 domain (E, F). Scale bar in (A) for (A–F) = 50 µm. (G–H′) Neural tube cells electroporated with Shh at HH stage 12 induce the expression of the p3 marker Nkx2.2 (H′) but not the FP expressed gene Arx (G) even at 72 hpt. Scale bar in (G, H) = 100 µm. (I) Early and late treatment with Shh generate comparable levels of Gli activity. (i1) An expression plasmid for Shh together with GBS-luc were electroporated either in the caudal preneural tube at HH stage 9 (blue) or in the neural tube at HH stage 12 (red) and Gli activity measured by luciferase assay at 24 hpt or at 48 hpt. (i2) Explants electroporated with the Gli reporter construct were cultured as indicated and luciferase activity measured. Incubation for 12 h with FGF did not elevate Gli activity (green arrow). (J–K′) Sustained expression of FGF8 eliminates FP differentiation in vivo. Overexpression of FGF8 at HH stage 8 and embryos cultured for 48 h. Scale bar in (J) for (J–K′) = 100 µm. (L) Transient expression of a gene of interest is achieved by a timed application of Tamoxifen, as assayed by a luciferase activity. Plasmids encoding 14×UAS-Luciferase and pCIG-ER-Gal4-VP16 were electroporated into HH stage 8 embryos, and 4-hydroxytamoxifen (4-OHT) was applied (red arrowheads) as described in Materials and Methods. At 8 h after the treatment, the embryos were washed thoroughly with HBSS (v; blue arrowhead). The luciferase assay was performed at the indicated time points, and the relative luciferase activities were calculated compared to control GFP electroporated embryos. (M–Z′) The transient overexpression of FGF signal promotes FP differentiation in the neural tube. (M) Schematic of the experiment. (N–Q′) Transient expression of HA-tagged constitutive-active MKK1 (HA-MKK1-SD) by electroporation of ER-Gal4-VP16 and 14×UAS-MKK1-SD at HH stage 8. Embryos were cultured for the indicated times. Expression of MKK1-SD was analyzed by immunohistochemistry for HA (for MKK1-SD; white in N, O, P, Q and red in N′, O′, P′, Q′) and GFP (as an electroporation control; green in N′, O′, P′, Q′). (R–Z′) The effect of the transient FGF signal on the expression of Pax6 (R, S, T, red in R′, S′, T′), Nkx2.2 (U, V, W, red in U′, V′, W′), and FoxA2 (X, Y, Z, red in X′, Y′, Z′) as analyzed by immunohistochemistry. The electroporated cells were identified with GFP (green in R′, S′, T′, U′, V′, W′, X′, Y′, Z′). At least four of the six embryos had similar phenotypes for each panel. Scale bar = 50 µm.
(TIF)

Figure S3 FGF and its downstream factors are required for the FP differentiation. (A) Schematic representation of the experiment. Expression plasmids containing either FGFR1ΔC or MKP3 were electroporated and explants prepared 3 hpt (−1 h). Explants were incubated in collagen for 1 h and then cultured in control medium for 3 h. For the inhibitor treatments, explants were prepared and cultured in the presence of 500 nM PD184352 or in 1 µM SSR128129E. (B) The expression of FGF target genes decreased rapidly in explants cultured in FGF signaling inhibitors. The expression levels of ETV5, IL17RD/SEF, and Nkx1.2 were examined by qRT-PCR. (C–G′) Images of Figure 2J–K′ at different magnifications. (C, D, E, F, G) are shown at 375 µm, and (D′, E′, F′, G′) are shown at 93.75 µm for each side. (H–P) FGF-receptor–mediated signaling is required for the FP differentiation. Explants electroporated with a dominant-negative version of FGFR1, FGFR1ΔC were prepared as in Figure 2G–M. Explants were cultured with 4 nM Shh for 48 h, and immunohistochemistry was performed with Arx (H, red in H′), Nkx2.2 (I, red in I′), and GFP (J, green in H′, I′). (K–L′) ETV5 is required for the FP differentiation. The dominant-negative version of ETV5, EnR-ETV5^DBD^, was electroporated at HH stage 8 and embryos were cultured for 48 h. The expression of Arx and Nkx2.2 was examined by immunohistrochemistry. Scale bar = 50 µm. (M–O) ETV5 is essential for the FP differentiation. The experiment was performed as in (H–J), and the expression of Arx and Nkx2.2 was analyzed. (P) Quantification of Arx- and Nkx2.2-expressing cells from explant experiments in Figures 2G–M and S2H–J,M–O. The data indicated with (†) are identical to those in Figure 2M. The population of Arx-positive cells is decreased also in the GFP-negative cells. This could be because (i) the transfected constructs had been transiently expressed and were not expressed at the time of the analysis, and/or (ii) homogenetic induction of FP results in cells that had differentiated into FP, thus inducing the differentiation of surrounding cells.
(TIF)

Figure S4 Nkx1.2 is expressed in the posterior neural plate and is regulated by FGF signaling. (A) Expression of Nkx1.2 in mouse embryos at e8.5, e9.5, and e10.5 analyzed by in situ hybridization. Scale bar = 0.5 mm for the e8.5 embryo and 1 mm for the e9.5 and e10.5 embryos. (B–D′) Overexpression of Nkx1.2 in [i] explants permits Arx induction by delayed Shh treatment, but many of the ectopic Arx-expressing cells are non-cell-autonomously induced. Explants were prepared from embryos electroporated with the Nkx1.2 expression plasmid and cultured as indicated in (B). The expression of Arx (C, red in C′), Nkx2.2 (D, red in D′), and GFP (green in C′, D′) were analyzed by immunohistochemistry. (E–K) Nkx1.2 promotes the ventralization of the neural tube and represses the expression of Pax6 and Irx3. Nkx1.2 was electroporated in the caudal preneural tube at HH stage 8, and embryos were incubated for 48 h and analyzed by immunohistochemistry for Nkx2.2 (F, red in F″), Pax6 (H, red in H″), and Irx3 (J, red in J″). Electroporated GFP-positive cells are shown in green in (F′, F″, H′, H″, J′, J″). Untransfected embryos were analyzed as a control (E, G, I). Scale bar in (E, F, G, H, I, J) = 50 µm. (K) Explants treated with FGF or electroporated with Nkx1.2 were incubated for 12 h and expression of the indicated genes analyzed by qRT-PCR. (L–P) The repressive effect of Nkx1.2 on Pax6 is not abrogated by RA signaling. Explants electroporated with control (L–L″) or Nkx1.2 (M–O″) were incubated with 10 nM RA for 24 h and Pax6 expression analyzed by immunohistochemistry. Pax6 expression was repressed in the Nkx1.2-expressing cells (M″, N″, O″) in a cell-autonomous manner. Quantification of Pax6 expression is provided in (P). Two different examples of Nkx1.2 expression are provided, one in which transfection happens to be restricted to half of the explant.
(TIF)

Figure S5 Analysis of the relationship between FGF signaling, RA, and Irx3. (A) FGF negatively regulates RA signaling. Retinoid activity in explants was assayed using a RARE (Retinoic Acid Responsive Element)-Luciferase reporter. Luciferase assays were performed after 12 h of incubation in control medium, 10 nM RA, or 5 nM FGF. Note that the bar graph is shown with a logarithmic scale. (B–C′) Attenuation of the Nkx1.2 function does not affect the expression of FGF8. Embryos electroporated with Nkx1.2^DBD^-VP16 (B, B′) or control GFP (C, C′) at HH stage 8 were cultured for 12 h and FGF8 expression assayed by in situ hybridization. Sections indicating FGF8 (b1, c1) and GFP (b2, c2) at the levels indicated by lines in (B) and (C). (D–I′) Irx3 and RA negatively regulate FP differentiation. Embryos electroporated with Irx3 or the constitutively active RAR (RAR-VP16) were cultured for 48 h. Expression of Arx (D, E, red in D′, E′), FoxA2 (F, G, red in F′, G′), Nkx2.2 (H, I, red in H′, I′), and GFP (green in D′, E′, F′, G′, H′, I′) assayed by immunohistochemistry. Scale bar (D) = 50 µm for (D–I′). (J–K′) Irx3 is a transcriptional repressor. Irx3^DBD^-EnR (J, J′) or Irx3^DBD^-VP16 (K, K′) were electroporated at HH stage 11 and embryos cultured for 24 h and analyzed by immunohistochemistry for Olig2 (J, K, red in J′, K′) and GFP (green in J′, K′). Scale bar (J, K) = 50 µm. (L) The combined attenuation of RAR activity and either Pax6 or Irx3 induces FP gene expression in explants. Explants electroporated with the indicated constructs were cultured in control medium for 12 h followed by 48 h culture in 4 nM Shh, as in Figure 4O. Nato3 and FoxJ1 expression were assayed by qRT-PCR.
(TIF)

Figure S6 FP and p3 identity can be generated in neural progenitor cells differentiated from mouse ES cells. (A) Schematic representation of the experiments. Shh was used at 2 µg/ml and RA at 30 nM. Treatment with Shh from day 3.5 to day 6 generated a large number of Arx-expressing cells (B), whereas treatment with RA from d3.0 induced the expression of Nkx2.2 but substantially less Arx (C). The expression of Arx (B, C, purple in B″, C″) and Nkx2.2 (B′, C′, green in B″, C″) was analyzed by immunohistochemistry. Scale bar (B) = 50 µm for (B–C″). (D, E) The expression of the indicated genes was analyzed by qRT-PCR in differentiated ES cells at d6.0 (D) and d3.5 (E). Expression levels of the genes in condition (ii) are presented relative to their levels in condition (i).
(TIF)

Figure S7 FoxA2 is an essential mediator of Arx expression independent of Shh signaling. (A) Control GFP or a dominant-negative FoxA2-EnR was electroporated and explants were prepared. Explants were cultured in 4 nM Shh for 48 h, and the expression of Arx and Nkx2.2 was analyzed by immunohistochemistry. (B–C″) Arx induction by FoxA2 is independent of Shh signaling. FoxA2 was electroporated with PtcΔ at HH stage 11 and embryos cultured for 48 h. Arx (B, red in B′, B″) expression was not blocked by inhibiting Shh signaling. Nkx2.2 (C, red in C′, C″) and GFP (B′, B″, C′, C″) expression was also assayed. Scale bar (B) = 100 µm. (D–F′) Irx3 inhibits the induction of Arx by FoxA2. Embryos electroporated with FoxA2 and Irx3 were cultured for 48 h. Arx (D, red in D′), Nkx2.2 (E, red in E′), Shh (F), and GFP (green in D′, E′, white in F′) expression was analyzed by immunohistochemistry (D–E′) or by in situ hybridization (F, F′) for the indicated genes. Sections of neural tube electroporated with a control GFP expression plasmid were also assayed for *Shh* expression (G). GFP expression is shown in (G′). Scale bar (D, G) = 100 µm. (H) Quantification of the Arx expression in the GFP-positive cells in Figure 5H–K′,S–T′.
(TIF)

Figure S8 Involvement of BMP4, FGF, and Nkx1.2 in the neural crest induction. (A–F′) Early and late exposure of neural progenitors to BMP has different effects on neural tube patterning. The expression plasmid carrying BMP4 was electroporated either at HH stage 9 or at HH stage 12 and embryos were cultured for 48 h. Immunohistochemistry for Sox10 (A, B, red in A′, B′), HNK1 (C, D, red in C′, D′), and Olig3 (E, F, red in E′, F′) was used to analyze NCC and dorsal interneuron induction. Migrating GFP-positive NCCs are indicated by arrowheads in (A, A′, C, C′). The dorsal midline is indicated by arrowheads in (F, F′). Scale bar (A–F) = 100 µm. (G–J) Images of Figure 6B′,C′,D′,E′ are at a lower magnification: 375 µm for each side. (K) The BMP activities at different developmental timing are similar. [i] explants electroporated with BRE-Luc were cultured in the indicated conditions and BMP signaling activity assayed. n.s., not significantly different. (L) Nkx1.2 on its own does not induce neural crest in vitro. [i] explants electroporated with or without Nkx1.2 were cultured for 12 h, and the expression levels of the indicated genes were analyzed by qRT-PCR. (M, M′) Overexpression of Pax6 in the roof plate inhibits the Snail2 expression. Pax6 was electroporated at HH stage 8 and embryos incubated for 12 h and analyzed by immunohistochemistry for Snail2 (M, red in M′). Scale bar = 50 µm. (N–P′) Transient FGF signaling allows the migration of the NCCs. Transient induction of FGF expression using the system described in Figures 1K–N′ and S2R–Z′ analyzed for Sox10 expression. Experimental conditions correspond to the schema in Figure S2M. Migrating NCCs are indicated by arrowheads and brackets. Scale bar (N, O, P) = 100 µm.
(TIF)

Table S1 Sequences of qRT-PCR primers.
(XLS)

Table S2 Expression values of Group A and Group B genes obtained from high-throughput RNA sequencing.
(XLS)

## Abbreviations

BMP: bone morphogenetic protein
EnR: drosophila Engrailed repressor domain
E.P.: electroporation
ER: estrogen receptor
ERK: extracellular-signal–regulated kinase
ES cells: embryonic stem cells
FGF: fibroblast growth factor
FP: floor plate
GR: glucocorticoid receptor
HH stage: Hamburger and Hamilton stage
hpt: hours posttransfection
MAPK: Mitogen-activated protein kinase
MKK: MAP kinase kinase
MN: motor neuron
NCC: neural crest cells
RA: retinoic acid
qRT-PCR: quantitative reverse transcription and polymerase chain reaction
Shh: Sonic Hedgehog
TGFβ: transforming growth factor beta
