# Supplementary figures and images for: Integration of Signals along Orthogonal Axes of the Vertebrate Neural Tube Controls Progenitor Competence and Increases Cell Diversity
Source: PLoS Biol. 2014 Jul 15;12(7):e1001907. doi: 10.1371/journal.pbio.1001907 (PMC4098999; doi:10.1371/journal.pbio.1001907)

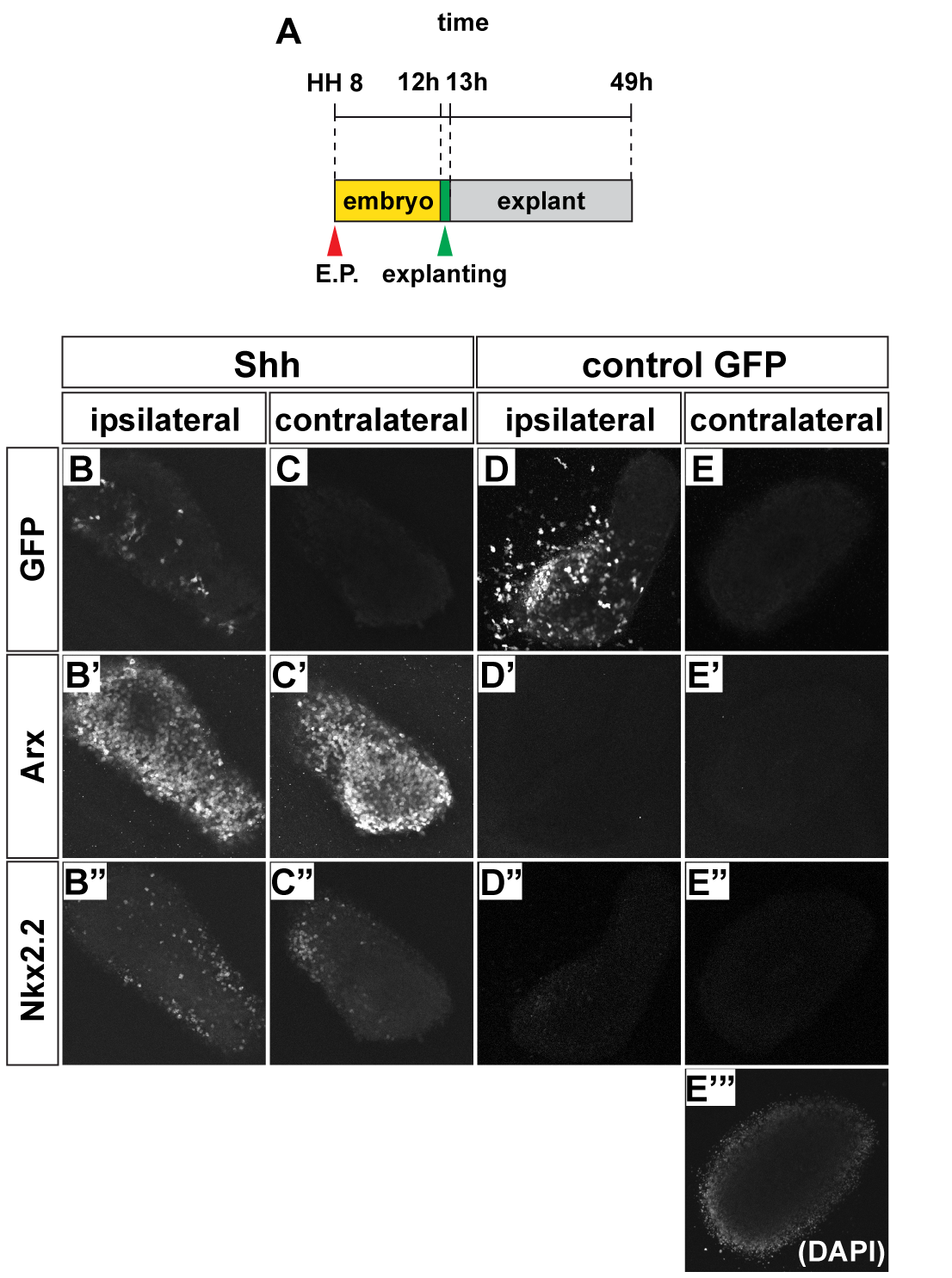

Supplement: Figure S1 — Transfected Shh affects the contralateral side of the neural tube. (A) Schematic representation of the experiment. pCX-Shh-N or control GFP was electroporated at HH stage 8, and embryos were cultured for 12 h. Explants were embedded in collagen drops and incubated for 1 h so that the collagen could harden. The explants were then incubated in the medium for an additional 36 h. (B–E″) Overexpressing Shh-N, but not GFP, ventralizes the intermediate neural explants not only on the ipsilateral but also on the contralateral side. Explants were analyzed by immunostaining for GFP (B, C, D, E), Arx (B′, C′, D′, E′), and Nkx2.2 (B″, C″, D″, E″) expression. Arx (C′) and Nkx2.2 (D′) expression was found in GFP-negative explants, suggesting that Shh-N spread contralaterally within 12 h of electroporation. DAPI staining in (E″′). (TIF) [file pbio.1001907.s001.tif]

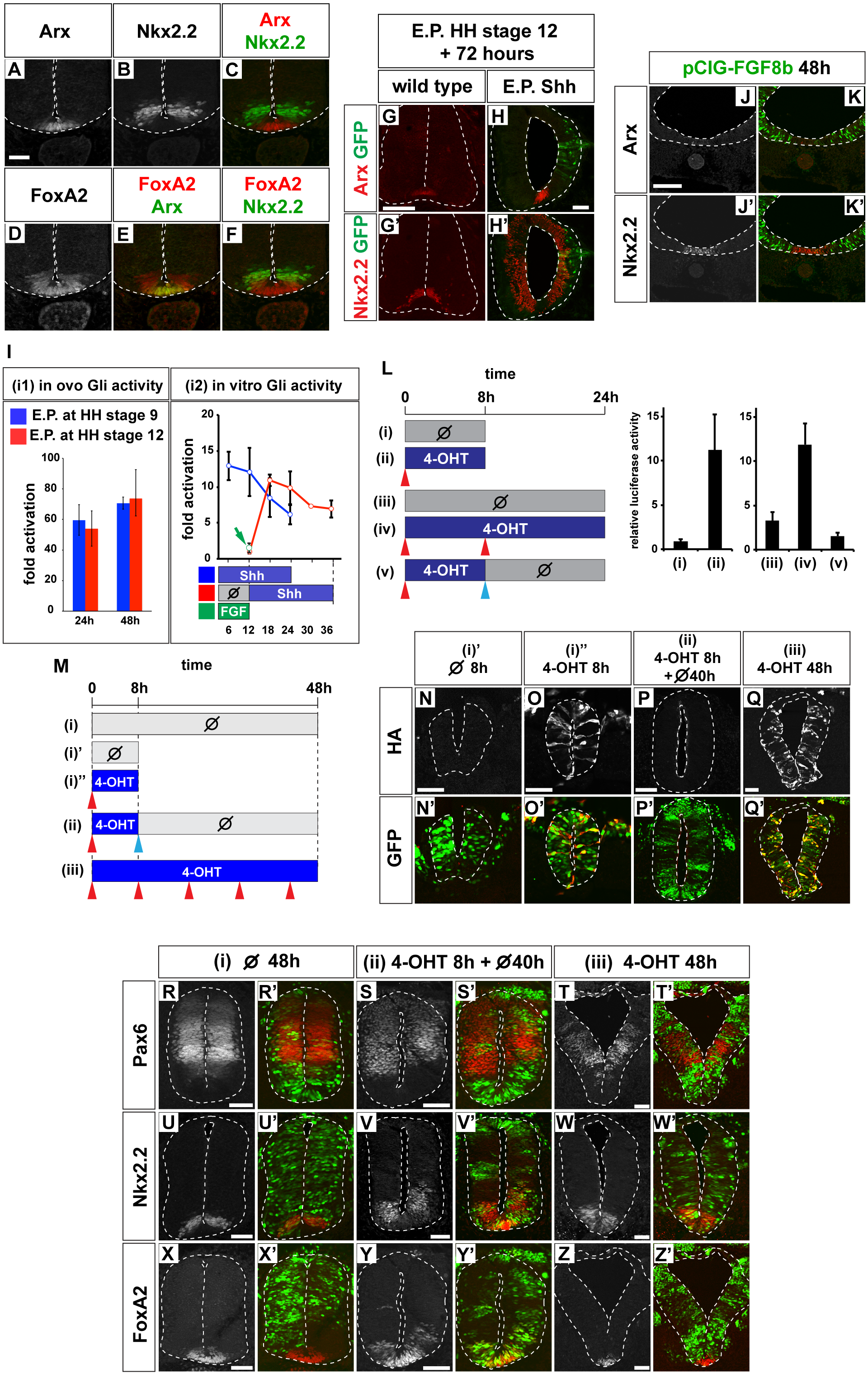

Supplement: Figure S2 — Expression of ventral neural genes and a regulatable expression system for the chick neural tube. (A–F) Expression pattern of Arx (A, C, E), Nkx2.2 (B, C, F), and FoxA2 (D, E, F) at HH stage 24. Arx and Nkx2.2 expression are mutually exclusive (C), whereas FoxA2 is expressed in the FP and the p3 domain (E, F). Scale bar in (A) for (A–F) = 50 µm. (G–H′) Neural tube cells electroporated with Shh at HH stage 12 induce the expression of the p3 marker Nkx2.2 (H′) but not the FP expressed gene Arx (G) even at 72 hpt. Scale bar in (G, H) = 100 µm. (I) Early and late treatment with Shh generate comparable levels of Gli activity. (i1) An expression plasmid for Shh together with GBS-luc were electroporated either in the caudal preneural tube at HH stage 9 (blue) or in the neural tube at HH stage 12 (red) and Gli activity measured by luciferase assay at 24 hpt or at 48 hpt. (i2) Explants electroporated with the Gli reporter construct were cultured as indicated and luciferase activity measured. Incubation for 12 h with FGF did not elevate Gli activity (green arrow). (J–K′) Sustained expression of FGF8 eliminates FP differentiation in vivo. Overexpression of FGF8 at HH stage 8 and embryos cultured for 48 h. Scale bar in (J) for (J–K′) = 100 µm. (L) Transient expression of a gene of interest is achieved by a timed application of Tamoxifen, as assayed by a luciferase activity. Plasmids encoding 14×UAS-Luciferase and pCIG-ER-Gal4-VP16 were electroporated into HH stage 8 embryos, and 4-hydroxytamoxifen (4-OHT) was applied (red arrowheads) as described in Materials and Methods. At 8 h after the treatment, the embryos were washed thoroughly with HBSS (v; blue arrowhead). The luciferase assay was performed at the indicated time points, and the relative luciferase activities were calculated compared to control GFP electroporated embryos. (M–Z′) The transient overexpression of FGF signal promotes FP differentiation in the neural tube. (M) Schematic of the experiment. (N–Q′) Transient exp [file pbio.1001907.s002.tif]

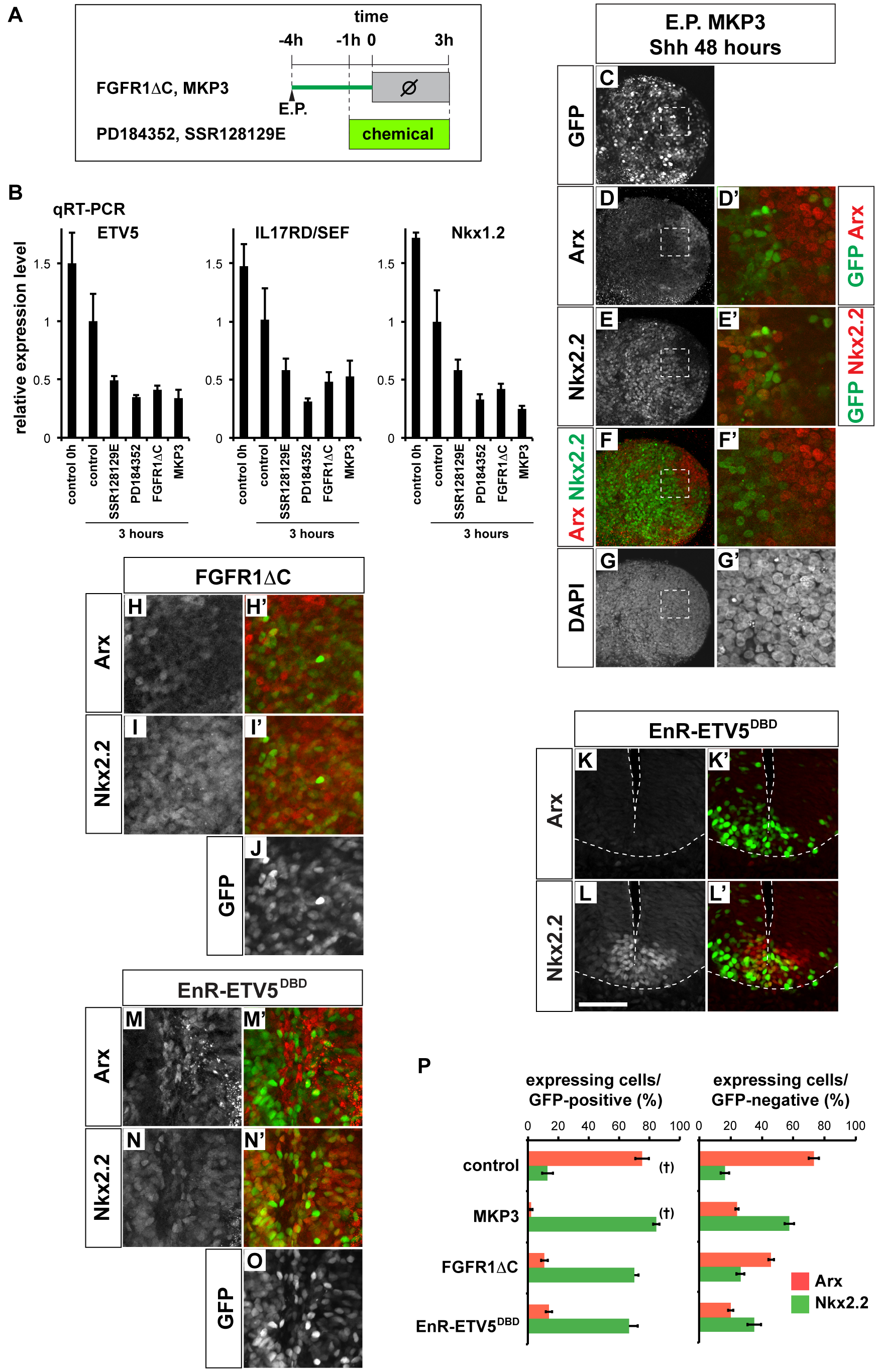

Supplement: Figure S3 — FGF and its downstream factors are required for the FP differentiation. (A) Schematic representation of the experiment. Expression plasmids containing either FGFR1ΔC or MKP3 were electroporated and explants prepared 3 hpt (−1 h). Explants were incubated in collagen for 1 h and then cultured in control medium for 3 h. For the inhibitor treatments, explants were prepared and cultured in the presence of 500 nM PD184352 or in 1 µM SSR128129E. (B) The expression of FGF target genes decreased rapidly in explants cultured in FGF signaling inhibitors. The expression levels of ETV5, IL17RD/SEF, and Nkx1.2 were examined by qRT-PCR. (C–G′) Images of Figure 2J–K′ at different magnifications. (C, D, E, F, G) are shown at 375 µm, and (D′, E′, F′, G′) are shown at 93.75 µm for each side. (H–P) FGF-receptor–mediated signaling is required for the FP differentiation. Explants electroporated with a dominant-negative version of FGFR1, FGFR1ΔC were prepared as in Figure 2G–M. Explants were cultured with 4 nM Shh for 48 h, and immunohistochemistry was performed with Arx (H, red in H′), Nkx2.2 (I, red in I′), and GFP (J, green in H′, I′). (K–L′) ETV5 is required for the FP differentiation. The dominant-negative version of ETV5, EnR-ETV5DBD, was electroporated at HH stage 8 and embryos were cultured for 48 h. The expression of Arx and Nkx2.2 was examined by immunohistrochemistry. Scale bar = 50 µm. (M–O) ETV5 is essential for the FP differentiation. The experiment was performed as in (H–J), and the expression of Arx and Nkx2.2 was analyzed. (P) Quantification of Arx- and Nkx2.2-expressing cells from explant experiments in Figures 2G–M and S2H–J,M–O. The data indicated with (†) are identical to those in Figure 2M. The population of Arx-positive cells is decreased also in the GFP-negative cells. This could be because (i) the transfected constructs had been transiently expressed and were not expressed at the time of the analysis, and/or (ii) homogenetic induction of FP results in cells that h [file pbio.1001907.s003.tif]

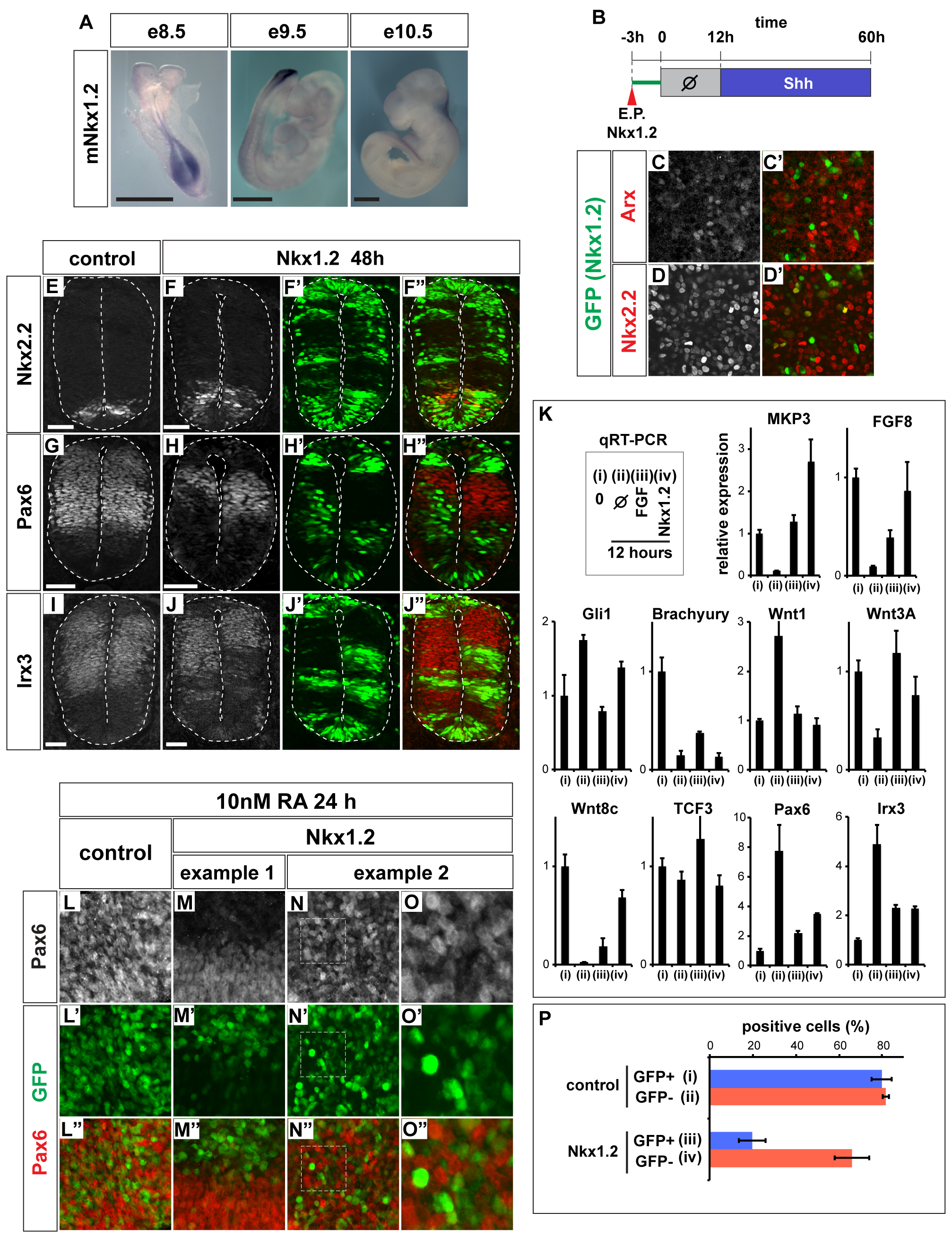

Supplement: Figure S4 — Nkx1.2 is expressed in the posterior neural plate and is regulated by FGF signaling. (A) Expression of Nkx1.2 in mouse embryos at e8.5, e9.5, and e10.5 analyzed by in situ hybridization. Scale bar = 0.5 mm for the e8.5 embryo and 1 mm for the e9.5 and e10.5 embryos. (B–D′) Overexpression of Nkx1.2 in [i] explants permits Arx induction by delayed Shh treatment, but many of the ectopic Arx-expressing cells are non-cell-autonomously induced. Explants were prepared from embryos electroporated with the Nkx1.2 expression plasmid and cultured as indicated in (B). The expression of Arx (C, red in C′), Nkx2.2 (D, red in D′), and GFP (green in C′, D′) were analyzed by immunohistochemistry. (E–K) Nkx1.2 promotes the ventralization of the neural tube and represses the expression of Pax6 and Irx3. Nkx1.2 was electroporated in the caudal preneural tube at HH stage 8, and embryos were incubated for 48 h and analyzed by immunohistochemistry for Nkx2.2 (F, red in F″), Pax6 (H, red in H″), and Irx3 (J, red in J″). Electroporated GFP-positive cells are shown in green in (F′, F″, H′, H″, J′, J″). Untransfected embryos were analyzed as a control (E, G, I). Scale bar in (E, F, G, H, I, J) = 50 µm. (K) Explants treated with FGF or electroporated with Nkx1.2 were incubated for 12 h and expression of the indicated genes analyzed by qRT-PCR. (L–P) The repressive effect of Nkx1.2 on Pax6 is not abrogated by RA signaling. Explants electroporated with control (L–L″) or Nkx1.2 (M–O″) were incubated with 10 nM RA for 24 h and Pax6 expression analyzed by immunohistochemistry. Pax6 expression was repressed in the Nkx1.2-expressing cells (M″, N″, O″) in a cell-autonomous manner. Quantification of Pax6 expression is provided in (P). Two different examples of Nkx1.2 expression are provided, one in which transfection happens to be restricted to half of the explant. (TIF) [file pbio.1001907.s004.tif]

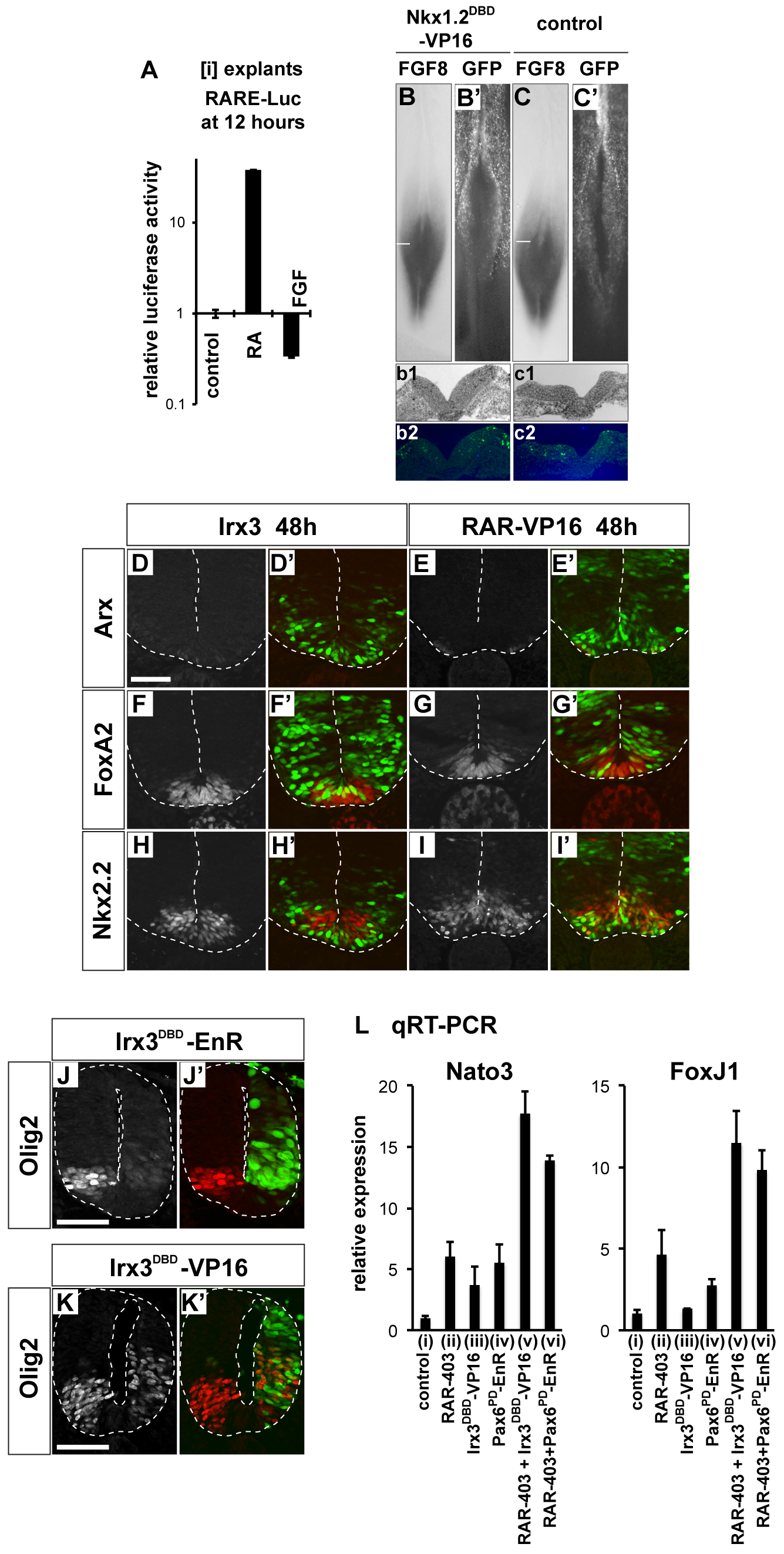

Supplement: Figure S5 — Analysis of the relationship between FGF signaling, RA, and Irx3. (A) FGF negatively regulates RA signaling. Retinoid activity in explants was assayed using a RARE (Retinoic Acid Responsive Element)-Luciferase reporter. Luciferase assays were performed after 12 h of incubation in control medium, 10 nM RA, or 5 nM FGF. Note that the bar graph is shown with a logarithmic scale. (B–C′) Attenuation of the Nkx1.2 function does not affect the expression of FGF8. Embryos electroporated with Nkx1.2DBD-VP16 (B, B′) or control GFP (C, C′) at HH stage 8 were cultured for 12 h and FGF8 expression assayed by in situ hybridization. Sections indicating FGF8 (b1, c1) and GFP (b2, c2) at the levels indicated by lines in (B) and (C). (D–I′) Irx3 and RA negatively regulate FP differentiation. Embryos electroporated with Irx3 or the constitutively active RAR (RAR-VP16) were cultured for 48 h. Expression of Arx (D, E, red in D′, E′), FoxA2 (F, G, red in F′, G′), Nkx2.2 (H, I, red in H′, I′), and GFP (green in D′, E′, F′, G′, H′, I′) assayed by immunohistochemistry. Scale bar (D) = 50 µm for (D–I′). (J–K′) Irx3 is a transcriptional repressor. Irx3DBD-EnR (J, J′) or Irx3DBD-VP16 (K, K′) were electroporated at HH stage 11 and embryos cultured for 24 h and analyzed by immunohistochemistry for Olig2 (J, K, red in J′, K′) and GFP (green in J′, K′). Scale bar (J, K) = 50 µm. (L) The combined attenuation of RAR activity and either Pax6 or Irx3 induces FP gene expression in explants. Explants electroporated with the indicated constructs were cultured in control medium for 12 h followed by 48 h culture in 4 nM Shh, as in Figure 4O. Nato3 and FoxJ1 expression were assayed by qRT-PCR. (TIF) [file pbio.1001907.s005.tif]

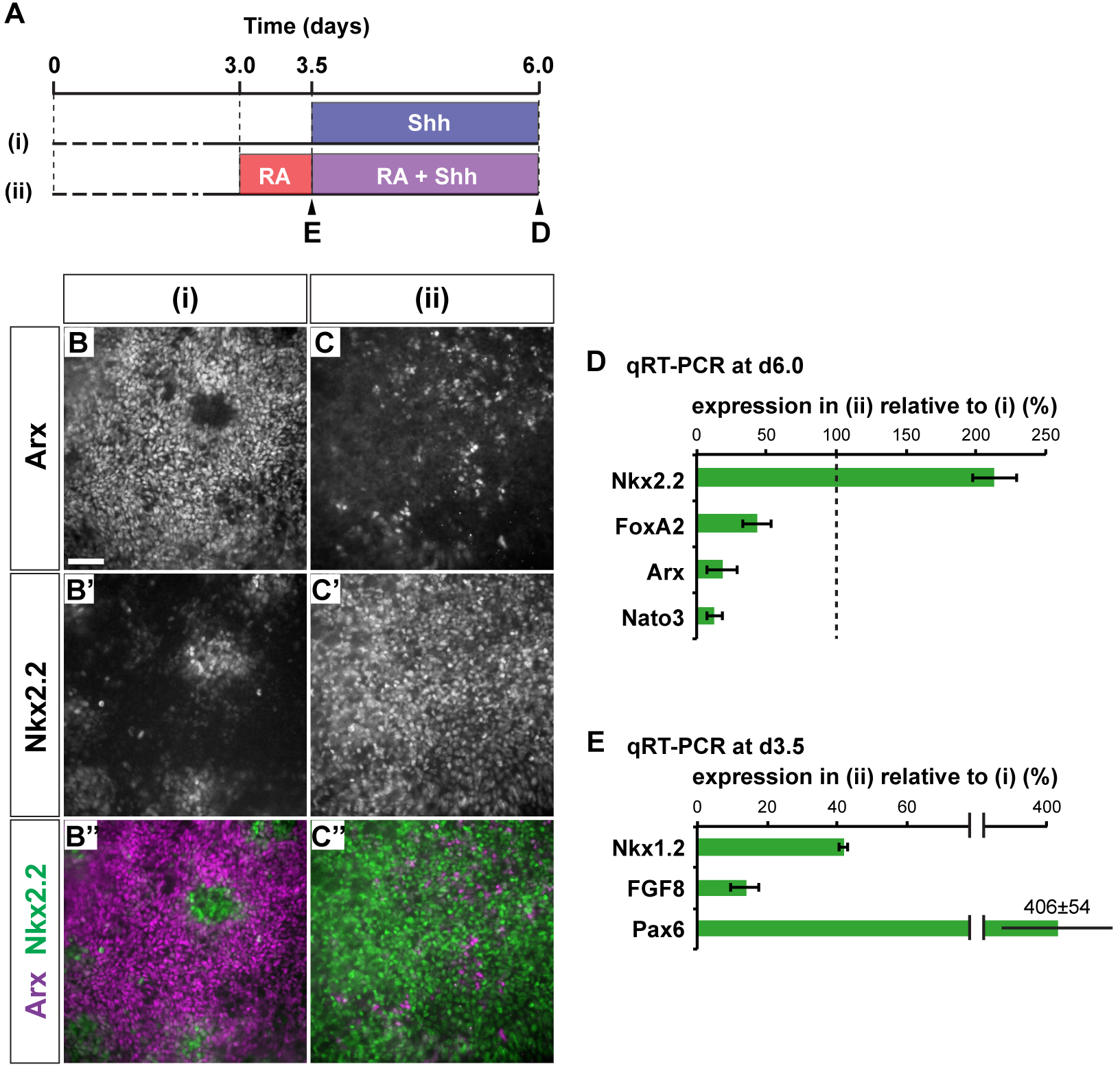

Supplement: Figure S6 — FP and p3 identity can be generated in neural progenitor cells differentiated from mouse ES cells. (A) Schematic representation of the experiments. Shh was used at 2 µg/ml and RA at 30 nM. Treatment with Shh from day 3.5 to day 6 generated a large number of Arx-expressing cells (B), whereas treatment with RA from d3.0 induced the expression of Nkx2.2 but substantially less Arx (C). The expression of Arx (B, C, purple in B″, C″) and Nkx2.2 (B′, C′, green in B″, C″) was analyzed by immunohistochemistry. Scale bar (B) = 50 µm for (B–C″). (D, E) The expression of the indicated genes was analyzed by qRT-PCR in differentiated ES cells at d6.0 (D) and d3.5 (E). Expression levels of the genes in condition (ii) are presented relative to their levels in condition (i). (TIF) [file pbio.1001907.s006.tif]

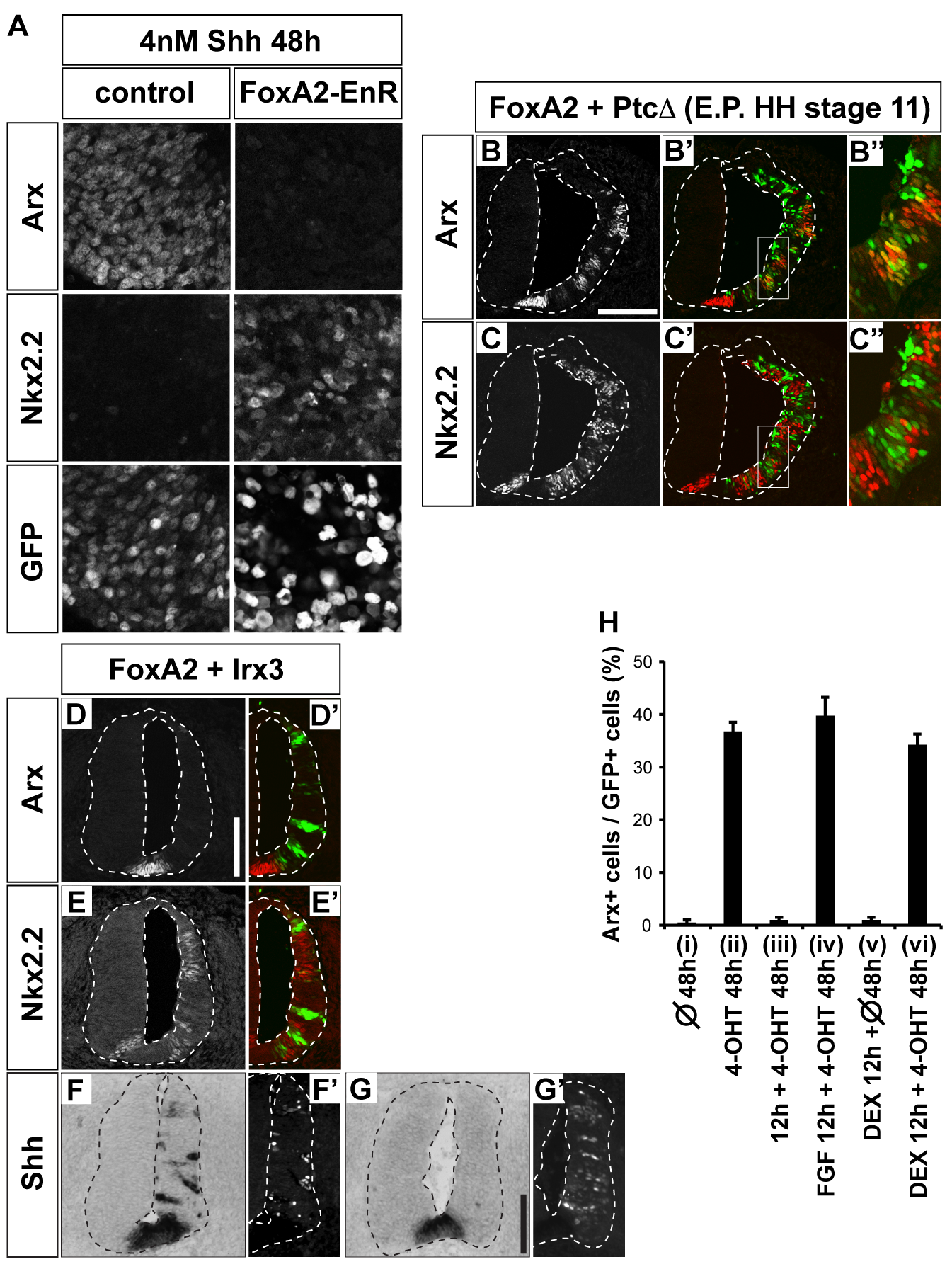

Supplement: Figure S7 — FoxA2 is an essential mediator of Arx expression independent of Shh signaling. (A) Control GFP or a dominant-negative FoxA2-EnR was electroporated and explants were prepared. Explants were cultured in 4 nM Shh for 48 h, and the expression of Arx and Nkx2.2 was analyzed by immunohistochemistry. (B–C″) Arx induction by FoxA2 is independent of Shh signaling. FoxA2 was electroporated with PtcΔ at HH stage 11 and embryos cultured for 48 h. Arx (B, red in B′, B″) expression was not blocked by inhibiting Shh signaling. Nkx2.2 (C, red in C′, C″) and GFP (B′, B″, C′, C″) expression was also assayed. Scale bar (B) = 100 µm. (D–F′) Irx3 inhibits the induction of Arx by FoxA2. Embryos electroporated with FoxA2 and Irx3 were cultured for 48 h. Arx (D, red in D′), Nkx2.2 (E, red in E′), Shh (F), and GFP (green in D′, E′, white in F′) expression was analyzed by immunohistochemistry (D–E′) or by in situ hybridization (F, F′) for the indicated genes. Sections of neural tube electroporated with a control GFP expression plasmid were also assayed for Shh expression (G). GFP expression is shown in (G′). Scale bar (D, G) = 100 µm. (H) Quantification of the Arx expression in the GFP-positive cells in Figure 5H–K′,S–T′. (TIF) [file pbio.1001907.s007.tif]

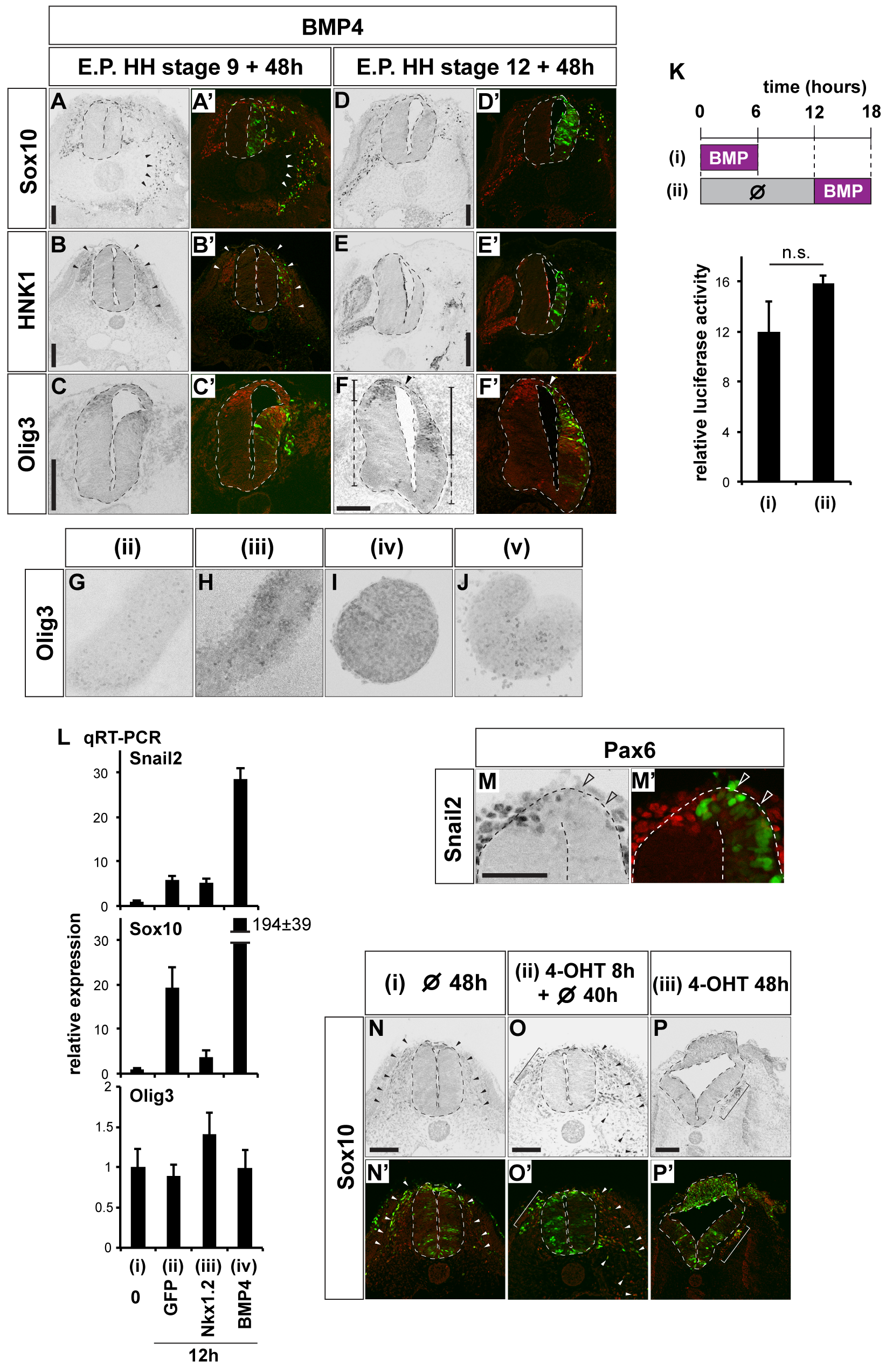

Supplement: Figure S8 — Involvement of BMP4, FGF, and Nkx1.2 in the neural crest induction. (A–F′) Early and late exposure of neural progenitors to BMP has different effects on neural tube patterning. The expression plasmid carrying BMP4 was electroporated either at HH stage 9 or at HH stage 12 and embryos were cultured for 48 h. Immunohistochemistry for Sox10 (A, B, red in A′, B′), HNK1 (C, D, red in C′, D′), and Olig3 (E, F, red in E′, F′) was used to analyze NCC and dorsal interneuron induction. Migrating GFP-positive NCCs are indicated by arrowheads in (A, A′, C, C′). The dorsal midline is indicated by arrowheads in (F, F′). Scale bar (A–F) = 100 µm. (G–J) Images of Figure 6B′,C′,D′,E′ are at a lower magnification: 375 µm for each side. (K) The BMP activities at different developmental timing are similar. [i] explants electroporated with BRE-Luc were cultured in the indicated conditions and BMP signaling activity assayed. n.s., not significantly different. (L) Nkx1.2 on its own does not induce neural crest in vitro. [i] explants electroporated with or without Nkx1.2 were cultured for 12 h, and the expression levels of the indicated genes were analyzed by qRT-PCR. (M, M′) Overexpression of Pax6 in the roof plate inhibits the Snail2 expression. Pax6 was electroporated at HH stage 8 and embryos incubated for 12 h and analyzed by immunohistochemistry for Snail2 (M, red in M′). Scale bar = 50 µm. (N–P′) Transient FGF signaling allows the migration of the NCCs. Transient induction of FGF expression using the system described in Figures 1K–N′ and S2R–Z′ analyzed for Sox10 expression. Experimental conditions correspond to the schema in Figure S2M. Migrating NCCs are indicated by arrowheads and brackets. Scale bar (N, O, P) = 100 µm. (TIF) [file pbio.1001907.s008.tif]
